# Advances in Biomarker Discovery for Early Ovarian Cancer Diagnosis

**DOI:** 10.3390/cells15211940

**Published:** 2026-10-25

**Authors:** Julia Niebora, Julia Spaczyńska, Aleksandra Partyńska, Natalia Penciak, Dominika Domagała, Krzysztof Data, Paul Mozdziak, Artur Bryja, Piotr P. Chmielewski, Paweł Dąbrowski, Teresa Wysocka, Maciej Zabel, Marzenna Podhorska-Okołów, Piotr Dzięgiel, Bartosz Kempisty, Ievgeniia Kocherova

**Affiliations:** 1 Division of Anatomy, Department of Human Morphology and Embryology, Faculty of Medicine, Wroclaw Medical University, 50-368 Wroclaw, Poland; julia.niebora@umw.edu.pl (J.N.); natalia.penciak@umw.edu.pl (N.P.); dominika.domagala@umw.edu.pl (D.D.); krzysztof.data@umw.edu.pl (K.D.); artur.bryja@umw.edu.pl (A.B.); piotr.chmielewski@umw.edu.pl (P.P.C.); pawel.dabrowski@umw.edu.pl (P.D.); 2 University College London Medical School, London WC1E 6DE, UK; julaspaczynska@gmail.com; 3 Division of Histology and Embryology, Department of Human Morphology and Embryology, Wroclaw Medical University, 50-368 Wroclaw, Poland; aleksandra.partynska@umw.edu.pl (A.P.); teresa.wysocka@umw.edu.pl or teresawysocka103@gmail.com (T.W.); maciej.zabel@gmail.com or maciej.zabel@umw.edu.pl (M.Z.); marzenna.podhorska-okolow@umw.edu.pl (M.P.-O.); piotr.dziegiel@umw.edu.pl (P.D.); 4 Prestage Department of Poultry Science, North Carolina State University, Raleigh, NC 27695, USA; pemozdzi@ncsu.edu; 5 Physiology Graduate Faculty, North Carolina State University, Raleigh, NC 27695, USA; 6 Institute of Medical Sciences, Adam Mickiewicz University, 64-920 Piła, Poland; 7 Department of Human Biology, Faculty of Physiotherapy, Wroclaw, University of Health and Sport Sciences, 51-612 Wroclaw, Poland; 8 Center of Assisted Reproduction, Department of Obstetrics and Gynecology, University Hospital and Masaryk University, 625 00 Brno, Czech Republic; 9 Department of Veterinary Surgery, Institute of Veterinary Medicine, Nicolaus Copernicus University in Torun, 87-100 Torun, Poland; 10 Center of Experimental Rheumatology, Department of Rheumatology, University Hospital Zurich, 8091 Zurich, Switzerland; ievgeniia.kocherova@usz.ch

**Keywords:** biomarkers, autoantibodies, ovarian cancer, miRNA, CA125, TP53, ctDNA

## Abstract

Ovarian cancer is one of the deadliest gynecological cancers worldwide. One promising therapeutic approach is the use of combined biomarkers. The Risk of Ovarian Malignancy Algorithm (ROMA) which combines CA125 with Human Epididymal Protein 4 (HE4) is an example of combining biomarkers to gain an optimal predictive outcome. Tumor Protein p53 (TP53) is a transcription factor with tumor suppressor properties. Mutations in TP53 constitute the most frequent genetic alterations observed in ovarian cancer. In contrast to conventional markers such as CA125, circulating tumor DNA (ctDNA) provides information about tumor mutations and insights into treatment response. Analysis of ctDNA enables the identification of tumor-associated mutations. Non-coding RNAs (ncRNAs) also influence cellular processes and act as effectors in oncogenic pathways by binding to RNA or proteins. One effector is Metastasis-Associated Lung Adenocarcinoma Transcript 1 (MALAT1), whose increased expression is associated with an aggressive tumor phenotype. MicroRNAs (miRNAs) are crucial in regulating the expression of genes associated with ovarian cancer development and progression. Exosomes carrying various molecules, including miRNAs and lncRNAs, contribute to ovarian cancer progression. Adipocytokines play an important role in the regulation of metabolic and inflammatory processes. Despite their diagnostic potential, the identified biomarkers require further validation.

## 1. Introduction

Ovarian cancer (OC) is one of the most lethal gynecological malignancies and represents the seventh leading cause of cancer-related mortality among women worldwide. Each year, more than 300,000 women are diagnosed with ovarian cancer and about 152,000 deaths are attributed to the disease [1]. In Poland, ovarian cancer ranks as the fifth most frequently diagnosed malignancy and fourth leading cancer-related death among women [2]. Early detection of ovarian cancer remains a major clinical challenge due to its asymptomatic nature until progression to advanced stages (II-IV) [1]. Ovarian cancer (EOC) is a heterogeneous disease characterized by molecular and histological diversity and accounts for about 90% of OC, with high-grade serous carcinoma (HGSOC) being the most prevalent and lethal subtype [3]. The absence of early clearly defined symptoms contributes to diagnosis at an advanced stage in more than 75% of patients. Consequently, prognosis remains poor, with a survival rate of approximately 30% ([1,4]). Although targeted therapies are available for selected patients, more than half of those treated experience disease recurrence within two years, highlighting the limited improvement in long-term survival. Early detection is therefore critical for improving patient outcomes, as it enables timely therapeutic intervention ([5]). Ovarian tumors are most commonly detected incidentally on ultrasound. Several sonographic features have been described as indicative of tumor malignancy. However, a substantial overlap exists between benign and malignant tumor appearances. In particular, some benign lesions have non-specific features, typically thin-walled, homogeneous, regular, and well-circumscribed characteristics, making accurate diagnosis challenging. On Doppler examination, benign lesions typically demonstrate absent or minimal vascularization, with normal flow and high resistance. Increasing structural complexity is associated with higher risk of malignancy, particularly in lesions exhibiting litho-cystic changes with thickened and irregular walls. Malignant lesions typically show low-resistance blood flow and a chaotic vascular distribution on Doppler imaging. Despite their clinical utility, these ultrasound-based methods require further optimization. Diagnostic accuracy could be improved through a more comprehensive analysis of tumor vascularization. In this context, contrast-enhanced ultrasound enables enhanced visualization of the microcirculation characteristic of ovarian tumors [6]. Histopathological examination remains the gold standard for ovarian cancer diagnosis, although it requires surgical excision of the tumor [1]. Improving the diagnosis of ovarian cancer and discovering novel molecular biomarkers could significantly improve treatment and patient prognosis [7]. Early detection of ovarian cancer through reliable biomarkers, along with characterization of the tumor genomic profiles, is critical for refining and personalizing new treatments [8]. Among the most established OC markers is tumor antigen 125 (CA-125), which has become the most widely used biomarker for ovarian cancer screening.. CA125 (MUC16) is a 3–5 million Dalton (Da) mucin peptide that promotes tumor cell proliferation and suppresses anti-tumor immune responses [9,10]. Although CA125 is elevated in almost 80% of advanced ovarian cancer cases, only approximately 50% of early-stage cases show CA125 elevation [8]. The clinical utility of CA125 has remained a subject of ongoing debate even though it has been employed for 40 years. Some studies suggest that CA125 plays a role in the initiation and progression of ovarian cancer. Despite its continued use in the development of targeted therapies due to its efficacy, research in clinical samples is limited by an incomplete understanding of the biochemical structure of this antigen [11]. Given the limitations of current biomarkers and critical importance of early detection, there is growing interest in identifying novel and more effective biomarkers for ovarian cancer. Such markers have potential to improve diagnosis and the efficacy of ovarian cancer therapies.

Promising new markers may enhance earlier detection and diagnosis as well as improve the effectiveness of current therapies and monitoring in ovarian cancer. In addition, emerging cell therapies offer the prospect of a less invasive and more precise approach to treating patients with ovarian cancer.

This literature review primarily identified studies by searching the PubMed database using a combination of keywords related to ovarian cancer, biomarkers, early diagnosis, prognosis, and specific molecular markers. Additional relevant publications were identified based on the reference lists of the selected articles. Studies were selected based on their relevance to the scope of the review. Particular attention was paid to studies addressing the diagnostic, prognostic, and biological significance of ovarian cancer biomarkers. Studies not directly related to ovarian cancer or the topics covered in this review were excluded. As this is a narrative review, no formal systematic review protocol or quantitative assessment of risk of bias was conducted.

## 2. Epidemiology and Clinical Characteristics of Ovarian Cancer

### 2.1. Clinical Evaluation of Therapeutic Strategies

Ovarian cancer is a complex disease posing a global health problem, being the seventh most common malignant tumor in women worldwide [4]. According to 2022 data from the Global Cancer Observatory (GLOBOCAN), there were 324,603 cases of ovarian cancer worldwide. Furthermore, it is estimated that the number of women diagnosed will increase by more than 55% by 2050, reaching 503,448 per year [12,13]. It is a heterogeneous disease with different subtypes, primarily epithelial, stromal, or germ cell in origin [14,15]. Ovarian cancer is characterized by late diagnosis, poor prognosis and variable survival rates, which may be due to the asymptomatic early stages of OC. Around 70% of ovarian cancer cases are diagnosed at an advanced stage, with metastases to adjacent organs or the peritoneal cavity. Ovarian cancer often spreads to the peritoneal cavity, affecting the intestines, liver, spleen and other internal organs. It can also metastasize to regional lesser pelvic and para-aortic lymph nodes [16]. According to the International Federation of Gynecology and Obstetrics (FIGO) classification system, the five-year overall survival rate decreases significantly as the stage of ovarian cancer progresses. For women diagnosed with stage I or II ovarian cancer, the average rate is between 74% and 93% [15,17]. However, according to the same classification, the rate is 40% to 50% for stage III cancer and only 20% to 30% for stage IV cancer. These statistics underscore the urgent need to detect ovarian cancer as early as possible [18,19]. Borderline ovarian tumors account for around 20% of EOC cases, which are characterized by a lack of basement membrane invasion and are usually diagnosed at an early stage. Early detection and treatment of ovarian cancer, with screening tests focusing on detecting invasive tumors are important techniques to further develop. Diagnosis of ovarian cancer (OC) is often challenging due to vague and non-specific symptoms. The main symptoms associated with advanced OC are bloating (77%), increased abdominal girth (64%), abdominal pain (22%), constipation (24%), back pain (45%), pelvic pain (26%), fatigue (34%) and frequent urination (16–34%) [15]. However, most early-stage ovarian cancer is asymptomatic, which significantly complicates diagnosis [18]. As many as 90% of ovarian cancer cases occur in postmenopausal women [20].

Ovarian cancer occurs at a rate of 1 in 2500 in the postmenopausal population making screening the entire population challenging. Therefore, it is highly justified to conduct screening for early-stage cancer in this group of women. In recent years, the focus has been on evaluating the effectiveness of CA-125 tumor antigen screening in serum, in conjunction with the Ovarian Cancer Risk Assessment (ROCA) algorithm. The analysis included both premenopausal and postmenopausal women. However, the results suggest that these tests are not fully effective. Due to the low incidence of this type of cancer in women at average risk, the tests produced a high number of false positives [21,22].

However, using the Risk of Ovarian Cancer (ROC) algorithm for multimodal screening at four-month intervals demonstrated significant changes in the detection of ovarian cancer in this family. Ovarian tumors are most often detected incidentally during routine ultrasounds. A diagnosis can be confirmed by sonography, computed tomography, or magnetic resonance imaging. Despite the morphological features described for ovarian tumors, there is still high similarity between benign and malignant lesions. Benign lesions are particularly problematic due to their lack of specificity; they are often heterogeneous and irregular. Ultrasound is used to distinguish between benign and malignant ovarian tumors. It also allows the histological type to be determined. This is important because the ovary is a common site for secondary tumors [23]. Doppler ultrasound is an ultrasound examination that allows for the assessment of blood flow in vessels. Benign lesions often show no or minimal blood supply, allowing them to be distinguished from malignant lesions. The blood supply to malignant lesions is irregular and shows chaotic vessel distribution. However, despite the clinical usefulness of ultrasound-based methods, there is a high margin of error, and ovarian cancer testing requires further optimization. Histopathological testing remains the gold standard, but it requires surgical intervention. The standard treatment is surgical cytoreduction, or the removal of tumor tissue, followed by chemotherapy. Therefore, it is crucial to search for new methods that are less invasive and more precise for detecting ovarian cancer. The search continues for new molecular biomarkers that would enable the early detection of this type of cancer, which could be crucial for developing targeted therapies [6,24].

The complex pathogenesis of ovarian cancer (OC) involves various histological subtypes and genetic predispositions. Moreover, a significant proportion of OC cases have a hereditary component [15]. According to the World Health Organization (WHO) classification of female genital cancers, there are five main histotypes of ovarian cancer (OEC), including high-grade serous ovarian cancer (HGSOC), low-grade serous ovarian cancer (LGSOC), mucinous ovarian cancer, endometrioid ovarian cancer, and clear cell ovarian cancer. Type I tumors usually have a good prognosis and grow slowly. These include low-grade serous carcinomas, borderline serous tumors, low-grade endometrioid tumors, clear cell carcinomas, and mucinous carcinomas. Type I tumors are usually detected at an early stage, but account for only 25% of ovarian cancers and 10% of ovarian cancer deaths. Type II tumors are aggressive, rapidly growing cancers that include highly malignant serous tumors and undifferentiated carcinomas. These tumors account for 75% of all ovarian cancers and 90% of ovarian cancer deaths. Due to their rapid growth and spread, they are more difficult to diagnose at an early stage. Additionally, mutations are present in 75–90% of cases [25,26]. Originally, it was suggested that ovarian carcinogenesis was the result of mutations accumulating in ovarian surface epithelial (mesothelial) cells. Repeated ovulations lead to epithelial damage, followed by reparative proliferation. During the repair process, metaplastic changes conducive to malignant transformation may occur [25]. However, it is now believed that high-grade serous ovarian cancer (HGSOC), the most common and aggressive form of ovarian cancer, originates in the fallopian tubes rather than the ovaries [15]. Epithelial cells of the fallopian tube, often as a result of squamous intraepithelial carcinoma, appear to be the origin of HGSOC with the metastasis spreading to the ovary. Current research focuses on understanding the molecular processes involved in ovarian cancer development and investigating potential chemotherapeutic agents. Despite advances in genomics and proteomics, effective, early-stage diagnostic tools remain absent, and population-wide screening remains challenging [27].

### 2.2. Preclinical Research

Among gynecological malignancies, ovarian cancer presents distinct diagnostic and therapeutic challenges due to its biological heterogeneity and the limited availability of reliable early-stage biomarkers underscoring the need for novel markers and innovative therapeutic strategies, which must be validated in preclinical and clinical studies.

Preclinical studies form the foundation for the identification of potential biomarkers. Both in vitro and in vivo models not only enable the analysis of tumor biology, but also allow systematic screening of body fluids for markers that could correlate with early disease development [28]. Both in vitro models, involving cell and organoid cultures, and in vivo models, such as xenograft mouse models or genetically engineered mouse models, can analyze the molecular mechanisms involved in disease pathogenesis and assess the dynamics of biomarker appearance in blood, peritoneal fluid or other tissues [29]. In addition, modern high-throughput technologies, such as proteomics, metabolomics and next-generation sequencing (NGS), allow comprehensive profiling of ovarian cancer at the molecular level, enabling the identification of markers with high sensitivity and specificity [30].

Mouse models play a key role in preclinical studies of EOC, enabling the study of disease mechanisms and the evaluation of new therapies. Orthotopic models are specifically designed to mimic the conditions of tumor growth and progression by implanting human tumor cells into a corresponding anatomical site in an experimental mouse. Through faithful in vivo reconstruction of the tumor microenvironment, including tissue structure, cellular interactions and vascularization, this approach enhances its value in cancer research. Orthotopic and metastatic tumor models also enable drug evaluation under more clinically relevant conditions. They account for target expression, drug distribution and interactions among tumor cells, vasculature and the host immune system. These models provide a comprehensive assessment of treatment response and closely mimic the course of a disease in a clinical setting. Therefore, they may be valuable for biomarker development and informing clinical trial decisions [31,32]. Additionally, Karakashev and Zhang reviewed the various xenograft (PDX) in mice where human tumor cells were implanted into immunocompromised mice models, and were found to have distinct advantages [29,33]. These models exhibit rapid tumor growth, allowing in vivo evaluation of therapies following in vitro studies. Low cost, high implantation efficiency and low variability in tumor size have made PDX models widely used in ovarian cancer research. Xenografts are used to investigate tumor initiation, growth, invasion, metastasis and therapeutic response. A key advantage of these models is the ability to use genetically engineered cell lines, enabling detailed study of the molecular mechanisms involved in EOC [29].

Another biologically relevant model in ovarian cancer research is the laying hen, which exhibits spontaneous carcinogenesis with a high incidence rate and close clinical and histologic resemblance to the human disease. In hens, ovarian tumors arise spontaneously, including epithelial ovarian cancer (EOC), the most common OC form in humans. Tumor lesions in hens resemble human ovarian cancer in progression and histopathologic features, making the avian model particularly useful for studying disease pathogenesis [34]. In addition, the chicken ovary shares structural and functional similarities with humans, allowing investigation of key cellular mechanisms in tumorigenesis. The hen model also enables the study of environmental influences on OC development in hens, which provides insights into disease progression [35,36]. Higher-order animal models have advanced our understanding of the genetic events leading to tumorigenesis and the cellular origin of ovarian cancer by recapitulating the tumor architecture and tumor microenvironment. The spontaneous development of OC in laying hens allows detailed study of genetic, biochemical, and environmental risk factors, as well as the processes of disease initiation and progression [37].

**Figure 1:**
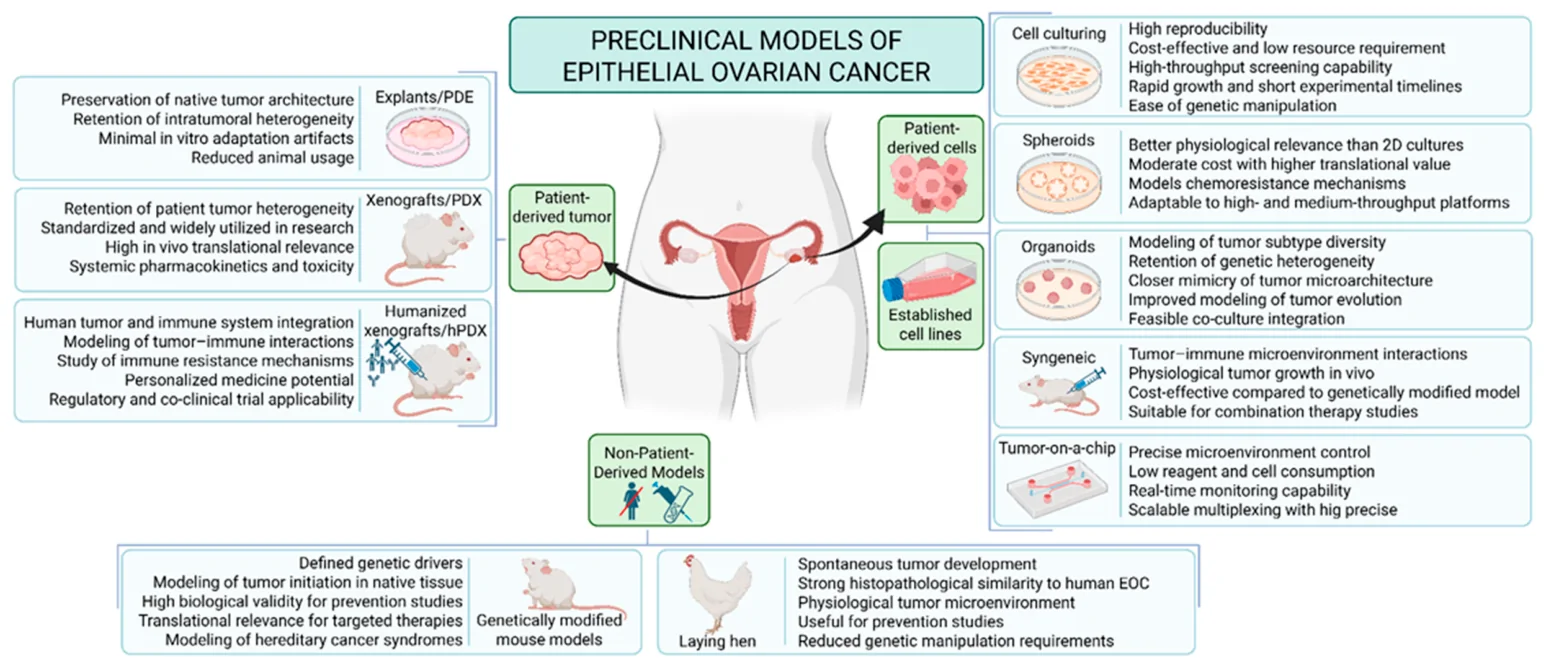
Graphical representation of common preclinical research models of epithelial ovarian cancer with main advantages (abb.: PDE, patient-derived explants; PDX, patient-derived explants).

Despite anatomical differences between hens and humans, significant similarities in EOC development have been shown (Figure 1). Laying hens undergo daily ovulation for at least two years, which increases their risk of ovarian cancer correlating with observations in women from developed countries, where frequent ovulation without fertilization correlates with a higher incidence of ovarian cancer. The hen model therefore supports the hypothesis that continuous ovulation is a driver of carcinogenesis. However, it should be noted that the absence of a luteal phase in hens and the associated progesterone deficiency may further increase the risk of developing the disease. Spontaneous carcinogenesis in the avian model also enables evaluation of chemopreventive compounds in EOC therapy [38]. Administration of Medroxyprogesterone acetate to laying hens has been shown to reduce ovulation frequency, effectively preventing the development of ovarian adenocarcinoma, underscoring the utility of the avian model in EOC [36]. In their study on ovarian cancer in laying hens, Ansenberger et al. (2009) [28] focused on E-cadherin. This transmembrane protein is responsible for maintaining connections between epithelial cells and enabling them to adhere together. The levels of E-cadherin in hens were compared to those in human ovarian cancer. The researchers found that E-cadherin levels were significantly higher in hens with cancer than in healthy hens. In humans, E-cadherin levels are reduced in many cancers, whereas in ovarian cancer, its expression levels are markedly elevated. The E-cadherin expression highlights the similarity between chicken models and humans. In ovarian cancer, E-cadherin properties may promote the colonization of metastatic sites by forming cell clusters that exhibit increased resistance to chemotherapy. Current research suggests that changes in E-cadherin levels occur during cancer progression, which may be significant during both tumor formation and the development of metastases. These findings imply that E-cadherin plays a pivotal role in the early detection of ovarian cancer [39,40].

Sahin et al. (2018) analyzed the chemopreventive properties of lycopene and its molecular mechanisms of action in ovarian cancer. Lycopene supplementation significantly reduced tumor incidence, number, and size, suggesting that its protective effects are mediated through antioxidant and anti-inflammatory mechanisms [41]. Another study evaluated the effects of two flax constituents—lignans and omega-3 fatty acids—on estrogen metabolism in a chicken model of ovarian cancer. These compounds exert anti-cancer effects by modulating 2-methoxyestradiol and SMAD7 levels and activating the p38 and ERK1/2 MAPK pathways [42]. Consistent with observations in humans, oviduct epithelium has been implicated in the development of spontaneous ovarian cancer in hens. Hen ovarian adenocarcinoma exhibits histological features, ascites, and peritoneal metastases comparable to advanced human ovarian cancer. Markers such as CA125, TP53, and E-cadherin demonstrate similar expression patterns. Employing multiple diagnostic markers is therefore useful not only for evaluating therapeutic interventions, but also for investigating molecular mechanisms of disease, including the role of the miR-200 family and E-cadherin in the development of inclusion cysts and ovarian [28,43]. Furthermore, studies show that the miR-200 family plays a significant role in forming inclusion cysts, and it is closely linked to regulating E-cadherin expression. Additionally, changes in their expression levels are associated with estrogen pathways, including TGF-β signaling, in both laying hens and humans. These findings highlight the importance of the chicken model in ovarian cancer research [29].

Although cross-species studies have revealed similarities in gene expression and molecular profiles between ovarian cancer in birds and humans, these findings should be interpreted with caution [44,45]. Comprehensive comparisons of mutational burden and copy-number changes across species are limited, and the extent to which specific genetic alterations and cellular origins are conserved is unclear [43,45]. Therefore, similarities in transcriptomic expression or biomarkers should not be interpreted as evidence of complete molecular conservation of ovarian cancer. Further comparative studies that integrate genomic, transcriptomic, and cellular data are needed to better define the extent of conserved oncogenic pathways across species.

Furthermore, quantitative data comparing treatment responses to spontaneous ovarian cancer in chickens with those observed in human ovarian cancer is limited. This limitation should be considered when evaluating the laying hen model’s prognostic and translational value [45,46,47].

## 3. Current Biomarkers for Ovarian Cancer

The development of non-invasive methods to detect and monitor cancer are still a significant challenge in modern oncology. Despite significant advances in this field, more precise and effective methods are needed to detect cancer in its early stages. Early detection of the disease is paramount, and less invasive testing methods can significantly improve patient care, quality of treatment, and minimize the risk of complications. For many years, researchers have been studying and evaluating biomarkers that could facilitate the diagnosis and treatment of ovarian cancer. In addition to well-established biomarkers such as CA125 and HE4, innovative approaches such as circulating tumor cells (CTCs), circulating tumor DNA (ctDNA), microRNAs, liquid biopsies, and extracellular vesicles are receiving attention [30,31,32]. Selected ovarian cancer biomarkers and their diagnostic and prognostic significance are summarized in Table 1.

**Table 1:** Ovarian cancer biomarkers.

Marker	Type	Histological Subtype(s) Reported in the Cited Studies	Role in Diagnosis/Prognosis	References
**CA125**	Protein	Mainly serous; histotype- dependent expression	Increased levels in ovarian cancer, endometriosis and inflammation.	[8,9]
**HE4**	Protein	Serous, endometrioid, clear cell and mucinous carcinomas; highest in serous and endometrioid	Differentiate benign from malignant tumors with CA125 [ROMA].	[33,34,35]
**NaPi2b**	Protein	Serous, endometrioid and mucinous epithelial ovarian carcinomas; expression varies by histotype	Overexpressed in 80–90% of ovarian cancers.	[36,37]
**lncRNA**	Long non-coding RNA	Various epithelial ovarian histotypes; histotype-specific expression	Regulator of gene expression; prognostic and diagnostic value.	[38]
**MALAT1** **[Metastasis-Associated Lung Adenocarcinoma Transcript 1]**	Long non-coding RNA	Serous and mucinous carcinomas; also investigated in other epithelial subtypes	Regulates proliferation, migration and invasion. Its overexpression is associated with resistance to chemotherapy.	[18,39,40,48,49,50,51]
**miRNA**	MicroRNA	Serous, endometrioid, clear cell and mucinous carcinomas; histotype-specific expression	Regulates signaling pathways involved in oncogenesis and affects the translation of mRNA into proteins.	[49,50,51,52,53]
**ctDNA [circulating tumor DNA]**	Cell-free DNA	Epithelial ovarian cancer; histotype not consistently specified	Early detection, assessment of prognosis and monitoring of response to treatment. Informs about the stage of the tumor and molecular changes in the tumor.	[54,55]
**Autoantibodies to TP53**	Autoantibodies	Predominantly serous; also detected in endometrioid, clear cell and mucinous tumors	May indicate mutations in the TP53 gene that are associated with cancer development. The TP53 protein is involved in the induction of apoptosis and DNA repair mechanisms.	[56]
**CXCL1 [C-X-C Motif Chemokine Ligand 1]**	Chemokine	Ovarian cancer; histological subtype not specified	Promotes angiogenesis and tumor cell migration. Elevated levels are associated with ovarian cancer progression.	[57]
**VEGF [Vascular Endothelial Growth Factor]**	Growth Factor	Epithelial ovarian cancer; histological subtype not specified	High expression in ovarian cancer is associated with poor prognosis.	[58]
**Adiponectin**	Adipocytokine	Ovarian cancer; histological subtype not consistently specified	Regulates glucose and lipid metabolism; may be correlated with the risk of disease.	[59,60]
**Leptin**	Adipocytokine	Ovarian cancer; histological subtype not consistently specified	Regulates energy metabolism. Elevated levels may be associated with cancer progression.	[60,61]
**Chemerin**	Adipocytokine	Ovarian cancer; histological subtype not consistently specified	Involved in inflammatory processes and metabolism. May affect angiogenesis and cancer cell migration.	[59,60]
**Omentin**	Adipocytokine	Ovarian cancer; histotype not reported	Has anti-inflammatory effects; may influence disease progression.	[62]

### 3.1. Cancer Antigen 125 (CA125)

CA125 is a glycoprotein encoded by the MUC16 gene on chromosome 19. It is overexpressed in over 80% of ovarian cancer patients and can be detected in the blood, allowing malignant ovarian tumors to be distinguished from other lesions [22,63]. In 2011, the UK National Institute for Health and Care Excellence (NICE) recommended CA125 as a screening test for women with suspected ovarian cancer [64,65]. CA125 remains the most extensively studied biomarker for monitoring OC progression. Mukama et al. (2022) evaluated 92 proteins in blood samples collected up to 18 months before an ovarian cancer diagnosis. Except for CA125, no markers provided clinically relevant diagnostic information, underscoring CA125’s effectiveness in detecting advanced ovarian cancer. However, CA125 has limited sensitivity for early-stage disease [66]. At Stage 1 or Stage 2, levels are elevated in only about 50% of patients with early-stage OC, limiting its utility as a standalone diagnostic marker. In contrast, CA125 blood concentrations/serum levels are usually markedly increased in advanced OC, making them useful for monitoring the effect of therapeutic treatment [1]. Funston et al. (2021) analyzed CA125 levels prior to diagnosis and found that patients with normal CA125 levels were more likely to be diagnosed at an early stage than those with elevated levels, suggesting that reliance on CA125 alone may delay diagnosis and worsen outcomes [65]. Furthermore, an elevated CA125 level is not specific to ovarian cancer as serum CA125 levels can be influenced by pregnancy, breast cancer and other factors; approximately 60% of individuals with elevated CA125 do not have OC [67]. Most high-grade serous ovarian cancers (HGSOC) are not detected at Stage 1, as these tumors tend to disseminate within a few months, as demonstrated in screening studies in high-risk women. The rapid progression of ovarian tumors complicates screening efforts in this group. Genetic predisposition is associated with earlier-onset, high-risk disease. Transvaginal sonography (TVS) and the protein biomarker CA125 are the most studied screening modalities, yet neither is adequate for population-wide screening [68,69].

### 3.2. HE4 (Human Epididymal Protein 4)

To improve early detection of ovarian cancer, researchers have investigated numerous potential biomarkers, both individually and in combination with CA125. Over 110 candidates have been studied both individually and in combination with CA125. In addition to CA125, other top candidates include HE4 (Human Epididymis Protein 4), transthyretin, CA15.3, and CA72.4 [22,70]. HE4 is a 124-amino acid glycosylated whey protein that constitutes a subfraction of one of the epithelial cell proteins of the epididymis. HE4 levels are elevated in approximately 60–75% of patients with ovarian cancer, compared to CA125 allowing for the identification of a small group of cases that CA125 does not detect. Although HE4 shows promising diagnostic potential, other biomarkers, such as CA15.3 and CA72.4, target different epitopes of the mucin MUC1 [31,70]. HE4 was originally isolated from the epididymis and may play a role in sperm maturation. While weakly expressed in respiratory and reproductive epithelia, HE4 is overexpressed in ovarian tumors, especially in EOC [33,71]. The serum measurement of human HE4 is one of the most promising tests for early OC detection. However, it remains ineffective as a standalone marker for early detection [71].

Several algorithms have been developed to improve ovarian cancer diagnosis. Among these, the Risk of Ovarian Malignancy Algorithm (ROMA) integrates HE4 and CA125 levels with menopausal status to stratify patients into high- and low-risk groups. A study evaluated the diagnostic performance of serum HE4, CA-125 and the ROMA index, finding that marker levels are highly variable in premenopausal women, reducing diagnostic reliability. In postmenopausal women, the diagnostic value of the markers improves due to the absence of menstrual cycles, and ROMA demonstrates increased sensitivity and specificity for early detection of ovarian cancer [72].

Although the ROMA demonstrates high sensitivity 94% for predicting EOC in women with pelvic tumors, its specificity is only 75%. The HE4 biomarker alone has been found to outperform ROMA [34]. Studies show that HE4 is effective as a clinical biomarker for early-stage ovarian cancer detection. Some studies suggest that the serum HE4 level is elevated in nearly 92% of ovarian cancer patients, indicating similar sensitivity and higher specificity than CA125 [35,73]. Compared to CA125, HE4 is less frequently elevated in benign gynecological conditions, such as endometriosis and uterine fibroids, suggesting that it is more specific. Unlike prostate cancer, where a definitive biomarker such as prostate-specific antigen (PSA) greatly aids diagnosis and reduces healthcare costs, no single biomarker currently provides such clear diagnostic guidance for ovarian cancer [73]. Nevertheless, HE4 has been shown to be more sensitive than CA125 in detecting early-stage ovarian cancer [72,74]. Interestingly, HE4 may also have prognostic potential in addition to diagnostic value, helping to identify patients at risk of ovarian cancer [34,75]. When combined with CA125, HE4 appears to have greater prognostic value for OC than either marker alone [76]. However, the diagnostic and prognostic value of CA125 and HE4 should be interpreted in the context of tumor subtype and stage. Although these biomarkers are commonly used to diagnose and predict the prognosis of ovarian cancer, their effectiveness may vary depending on the histological subtype CA125 sensitivity, for example, may be lower in less common subtypes, such as clear cell and mucinous carcinomas, but is particularly high in high-grade serous ovarian cancer (HGSC). Therefore, findings derived predominantly from HGSC should not be generalized to all ovarian cancer subtypes [77,78].

Comparative analyses of CA125 and HE4 markers, as well as models based on biomarker combinations, revealed that diagnostic value of multi-marker approaches is influenced by the clinical characteristics of the study population. Meta-analysis of 32 studies and reported AUC values of 0.87 for CA125, 0.89 for HE4, and 0.91 for ROMA. HE4 demonstrated higher specificity (93.6%) than CA125 (82.1%) and ROMA (82.4%). The authors accounted for heterogeneity related to menopausal status, FIGO stage, detection method, and study design. They found that CA125 and ROMA performed better in postmenopausal women than in premenopausal women. These results suggest that the potential benefits of combining biomarkers should be interpreted in the context of a given population and diagnostic conditions rather than being considered universally superior to individual biomarkers [79]. The diagnostic accuracy of HE4 and CA125 markers may depend on the characteristics of the study population and the diagnostic threshold used. In a systematic review and meta-analysis of 17 studies involving 3404 patients with pelvic masses, found that HE4 had a sensitivity of 79.4% and a specificity of 84.1%, compared to 81.4% and 56.8%, respectively, for CA125. Importantly, the predictive values varied depending on the prevalence of ovarian cancer in the study population. At a prevalence of 40%, the positive predictive value (PPV) was 76.9% for HE4 and 55.6% for CA125. At a prevalence of 15%, meanwhile, the negative predictive values (NPVs) were 95.8% and 94.4%, respectively. These results highlight that the clinical utility of ovarian cancer biomarkers must be evaluated alongside the characteristics of the target population, as differences in disease prevalence and diagnostic cut-off values can significantly impact their predictive value [80]. Thus, differences in patient selection, disease prevalence, menopausal status, disease stage, and biomarker cutoff values can substantially impact the reported diagnostic performance of individual and combined biomarker models.

### 3.3. Autoantibodies

Autoantibodies against mutated proteins can be induced in small ovarian or fallopian tube tumors. Autoantibodies are more sensitive and can detect the disease at an earlier stage than CA125 Autologous antibodies can target mutated TP53 (Tumor Protein TP53), a transcription factor with tumor suppressor properties central to apoptosis and DNA repair mechanisms. Mutations in TP53 are the most common genetic alterations in ovarian cancer, and mutations are observed in 96% of high-grade serous carcinoma cases [18]. With a specificity of 97%, autoantibodies were detected in 21–30% of serum samples from ovarian cancer patients. Of the 164 cases with elevated CA125, 20.7% had elevated TP53 autoantibodies [56,81]. Among 34 ovarian cancer cases detected by the Risk of Ovarian Cancer Algorithm [ROCA] screening algorithm, TP53 autoantibody titers were elevated on average 8 months prior to increases in CA125 levels. In contrast, in the 9 cases missed by ROCA, autoantibody titers were elevated 22.9 months before the increase in CA125. These results strongly suggest that TP53 autoantibody levels may provide an earlier indicator of ovarian cancer than CA125, and represent the first biomarker to demonstrate a clinically significant lead time over CA125. Recent studies confirm the potential of anti-p53 autoantibodies as a marker for the early detection of ovarian cancer. These studies also highlight the possibility of detecting autoantibodies prior to a medical diagnosis, suggesting their potential use in screening alongside CA125 and HE4. However, current knowledge of the markers and their utility remains limited requiring further research in clinical settings [18,82].

## 4. Emerging Molecular Biomarkers and Therapeutic Targets in Ovarian Cancer

### 4.1. Liquid Biopsy Approaches in Ovarian Cancer

#### 4.1.1. Circulating Tumor DNA (ctDNA)

**Figure 2:**
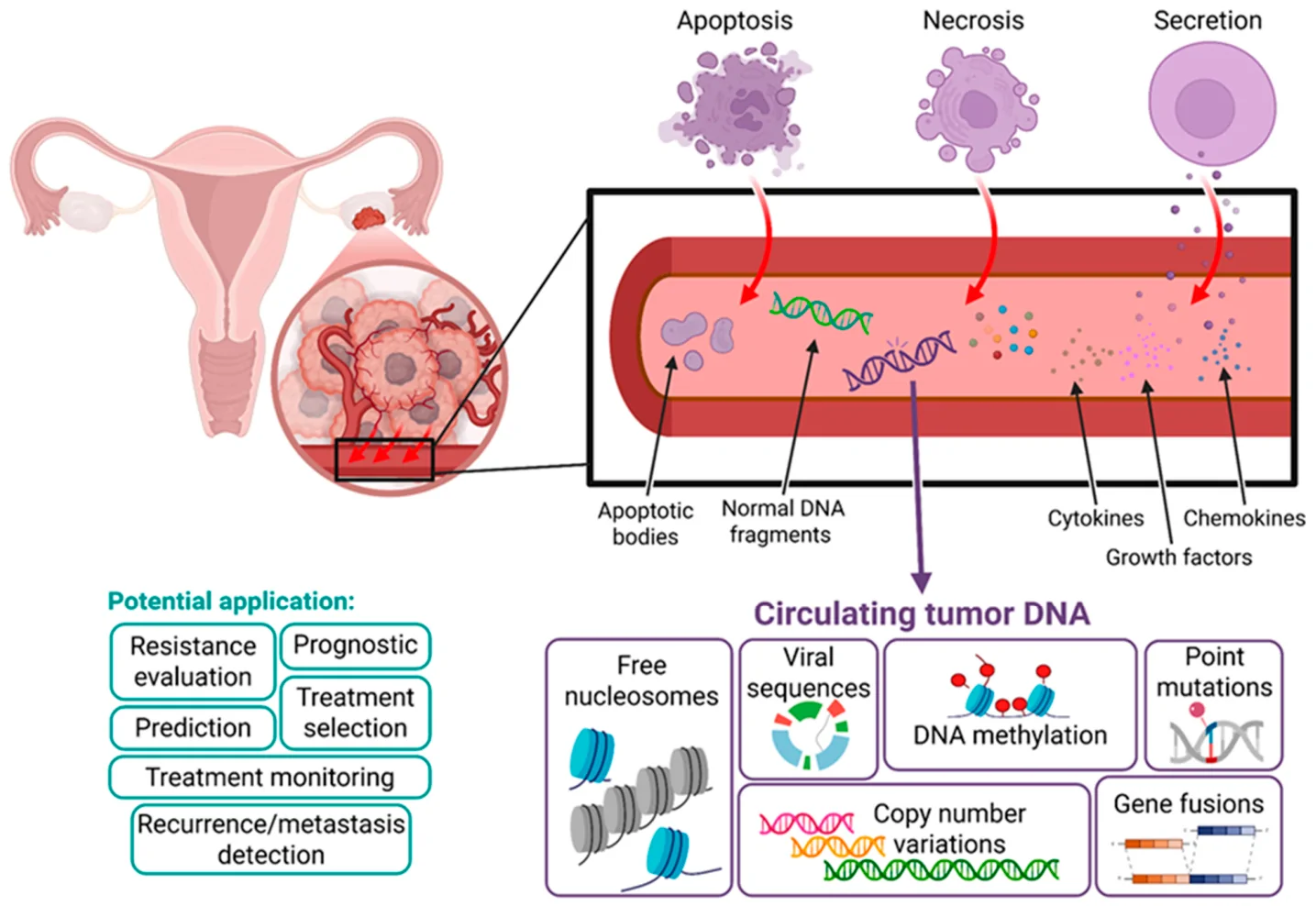
The possible sources of circulating tumor DNA (ctDNA) in vessels and the types of pathological changes counted as ctDNA. The potential uses of ctDNA in clinical and preclinical practice are also highlighted.

Identification and characterization of tumor-derived components are essential for biomarker discovery. These components may include circulating tumor cells (CTCs), exosomes, and circulating DNA (ctDNA). In recent years, circulating tumor DNA has emerged as one of the most promising biomarkers for early cancer detection, as tumors secrete ctDNA into the bloodstream before they become detectable on imaging or clinical symptoms appear [55,83]. ctDNA enters the circulation via apoptosis, tumor necrosis or circulating tumor cells. Liquid biopsy and ctDNA analysis correlate closely with the mutational profile of solid tumors. ctDNA levels are higher in patients with advanced breast, colorectal, or esophageal cancer compared to those in the early stages [54]. In ovarian cancer, preclinical and clinical studies increasingly support ctDNA’s role in diagnosis, prognosis, and prediction of treatment response (Figure 2) [54]. Liquid biopsy is a minimally invasive procedure that detects molecular biomarkers in body fluids, avoiding invasive and expensive procedures [84]. As a safer alternative to surgical biopsy, liquid biopsy can provide substantial information about tumor biology from a simple blood sample. Importantly, liquid biopsy allows longitudinal monitoring of tumor cells through analysis of circulating tumor cells (CTCs), extracellular microvesicles, and ctDNA [85]. Kumar et al., 2018 [86], suggested that ctDNA analysis supported tumor characterization and monitoring of treatment response. A growing body of evidence suggests that ctDNA has substantial potential as a highly sensitive biomarker for monitoring response to multiple treatments, including targeted therapies. However, further research is needed to tailor ctDNA-based therapies to the individual clinical characteristics of ovarian cancer patients [87,88].

However, the use of circulating DNA has its limitations. Circulating tumor DNA (ctDNA) is constantly released into the bloodstream, partly due to the increased apoptosis of hematopoietic cells with age [89,90]. Older individuals’ blood cells can acquire numerous somatic mutations that lead to clonal hematopoiesis of indeterminate potential (CHIP). These mutations may involve the TP53 gene, alterations of which are frequently observed in high-grade serous ovarian cancer [89,91]. Therefore, CHIP-associated mutations can lead to false-positive results and be misinterpreted as tumor variants in the TP53 gene. Sequencing leukocyte DNA can effectively identify and filter out CHIP-associated variants [89,91].

Another limitation of circulating tumor DNA (ctDNA) testing may be the tumor microenvironment, tumor stage and location, and cancer cell proliferation and apoptosis rates. The detectability of circulating tumor DNA (ctDNA) increases in advanced stages of cancer. However, its detection in early stages may be limited by factors such as low tumor mass [90,92]. The tumor microenvironment can influence the release of ctDNA into the bloodstream. Additionally, biological and physical barriers may limit ctDNA entry into the circulation. In the early stages of disease, ctDNA may account for only approximately 0.01% of total circulating DNA [89,90]. Low levels of ctDNA can lead to false-negative results in patients with active cancer. Therefore, using additional molecular markers, such as DNA methylation patterns, may increase the sensitivity and specificity of detection [90,91].

Other limitations include differences among detection platforms, sequencing depth, and the range of analyzed mutations. Panels that target specific genes may not detect variations outside the analyzed range, which can affect the interpretation of results. Additionally, preanalytical factors, such as time from blood collection to analysis, temperature, storage conditions, sample volume, surgical procedures, and comorbidities, can influence circulating tumor DNA (ctDNA) analysis results. These factors can lead to false-positive or false-negative results [90,93]. These limitations should be considered when evaluating the clinical potential of circulating tumor DNA (ctDNA) for early ovarian cancer detection, treatment response monitoring, and recurrence prediction.

#### 4.1.2. Exosome-Derived Biomarkers

Exosomes, which carry miRNAs, lncRNAs, and other bioactive molecules, play an important role in ovarian cancer progression. Cancer cells secrete higher levels of exosomes than healthy cells, and these vesicles contain a variety of molecules that modulate the tumor microenvironment and processes such as metastasis and treatment resistance. In ovarian cancer, exosomes carry miRNAs that can modulate the expression of genes associated with metastasis and drug resistance, which is particularly relevant in the context of advanced disease when therapy is less effective [94,95]. Understanding the role of ncRNAs and exosomes in ovarian cancer may reveal new diagnostic and therapeutic avenues in the treatment of gynecological cancers [96].

Additionally, the presence of exosomes and their contents can provide information about the condition of the donor’s cells. Their composition is highly specific to a particular cell type. The RNA and molecular cargo inside these vesicles are protected by a lipid membrane, contributing to their stability; this increases their detectability in most body fluids, such as peritoneal fluid, blood, and urine. The high stability and detectability of exosomes in fluids enhances their significance in the context of liquid biopsies, increasing the likelihood of less invasive tumor progression monitoring. Despite growing interest, the use of exosomes and knowledge of their isolation methods remain challenging. Current research focuses on capturing exosomes and evaluating the diagnostic potential of their molecular cargo, which primarily consists of microRNAs (miRNAs), long non-coding RNAs (lncRNAs), and circular RNAs (circRNAs). Additionally, innovative methods for capturing and isolating them are still being developed and could significantly contribute to their application in clinical trials [97].

Some researchers believe that these vesicles target specific cell types. One type of exosome, derived from cancer cells, has been found to be taken up by stem cells but not by other cell types [98]. This type of selectivity in their capture allows them to be used in many ways. For example, they can activate the immune system. When derived from cancer cells, they can stimulate tumor growth within and beyond the tumor itself. Additionally, numerous studies demonstrate their effectiveness as therapeutic carriers in treating various types of cancer, including breast [99,100] prostate [99] and lung cancers [101].

Recent studies indicate that, in addition to their role in intercellular communication, extracellular vesicles may also be involved in interactions between ovarian cancer cells and the tumor-associated nervous system, which may contribute to tumor progression and treatment resistance. In high-grade serous ovarian cancer (HGSOC), dense tumor innervation is associated with tumor-derived small extracellular vesicles that may stimulate neurite growth. Experimentally removing nerves that infiltrate tumors reduced tumor growth and increased sensitivity to carboplatin, suggesting a potential role for tumor–nerve interactions in platinum resistance [102].

Remodeling of the neural network may contribute to the formation of a metastatic microenvironment as the tumor spreads within the peritoneal cavity. This includes interactions with ascites fluid, adipose tissue, immune cells, and stromal components [103].

Extracellular vesicles may contribute to the spread of cancer in the peritoneal cavity by transporting molecules between ovarian cancer cells and cells of the peritoneal microenvironment. For example, it has been shown that extracellular vesicles derived from ovarian cancer and containing MMP1 mRNA promote damage to mesothelial cells and the spread of cancer within the peritoneal cavity, and vesicles containing MMP1 mRNA have been detected in malignant ascites [104].

These observations confirm the possibility of linking molecular signals associated with extracellular vesicles to non-coding RNA biomarkers. The potential use of microRNA (miRNA) profiles associated with extracellular vesicles (EVs) derived from malignant peritoneal fluid and plasma as diagnostic markers in ovarian cancer was examined [105].

Furthermore, proteins associated with extracellular vesicles (EVs) and long non-coding RNAs (lncRNAs) have been linked to platinum resistance in high-grade serous ovarian cancer (HGSOC), suggesting that EVs may provide additional information useful for predicting treatment response [106,107].

Nevertheless, the direct relationship between tumor innervation and circulating non-coding RNAs or circulating tumor DNA remains largely unexplored. Therefore, combining neuronal features with molecular biomarkers associated with extracellular vesicles and circulating molecules may represent a promising direction for future research aimed at improving the prediction of peritoneal dissemination, ascites development, and platinum resistance.

### 4.2. Non-Coding RNAs in Ovarian Cancer

Non-coding RNAs (ncRNAs) play a significant role in ovarian cancer pathogenesis. As with other malignant tumors, ncRNAs influence the regulation of numerous cellular processes, including proliferation, apoptosis, and migration. The most important ncRNAs are microRNAs (miRNAs), long non-coding RNAs (lncRNAs), and circular RNAs (circRNAs), which participate in complex regulatory processes. These ncRNAs can act as oncogenes or tumor suppressors, depending on the situation. Abnormal ncRNA expression is associated with disease initiation and progression, as well as the development of chemotherapy resistance. Furthermore, ncRNAs are considered highly promising prognostic and diagnostic markers. ncRNAs exhibit significant stability and widespread distribution throughout the body, making them potential targets for targeted molecular therapies in ovarian cancer [108].

#### 4.2.1. Long Non-Coding RNAs (lncRNAs)

**Figure 3:**
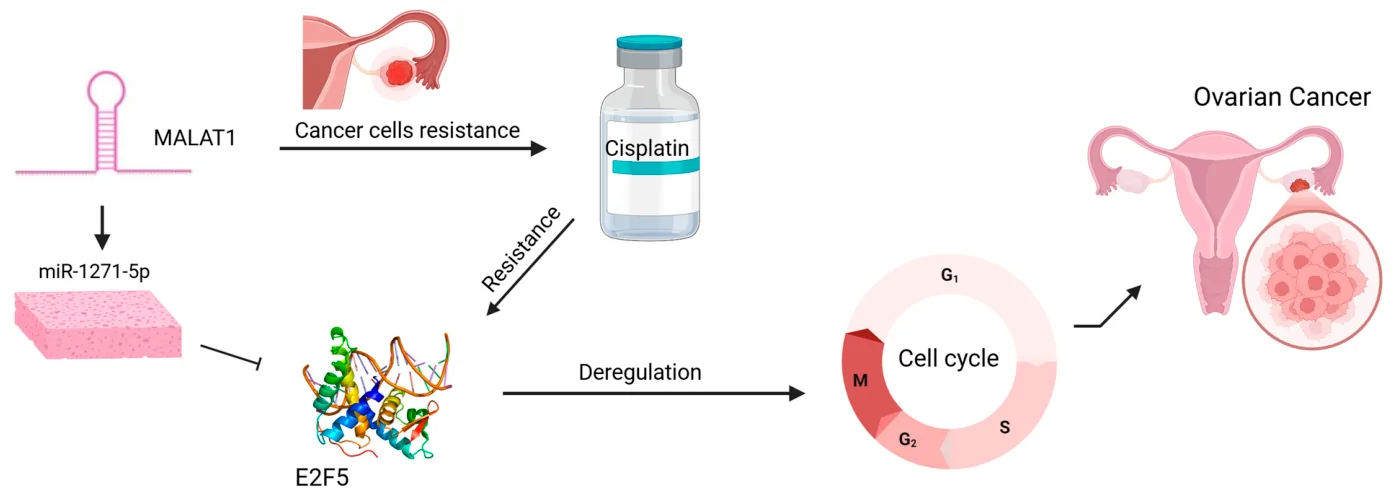
The role of long non-coding RNAs (lncRNAs) in ovarian cancer progression and cisplatin resistance.

LncRNAs act as gene regulators in ovarian cancer, influencing cell proliferation, apoptosis, cell migration, and metastasis. LncRNAs act as effectors within oncogenic pathways by binding to DNA, RNA, and proteins, leading to altered expression of genes involved in cell cycle control and cellular stress response. One example is MALAT1 (Metastasis-Associated Lung Adenocarcinoma Transcript 1), which is implicated in the development and progression of several cancers, including ovarian cancer. Elevated MALAT1 expression in ovarian cancer cells is associated with increased tumor aggressiveness and poor patient prognosis [109]. Studies suggest that MALAT1 contributes to resistance to cisplatin, a commonly used chemotherapeutic drug via miR-1271-5p/E2F5 axis. MALAT1 acts as a sponge for miR-1271-5p, leading to deregulation of E2F5, a family of transcription factors that play a key role in cell cycle regulation, cell proliferation, and differentiation. MALAT1 binds to miR-1271-5p and prevents its regulatory function. Since miR-1271-5p cannot downregulate E2F5, its expression increases. Increased E2F5 expression activates cell cycle genes, promoting cancer cell proliferation and chemoresistance (Figure 3) [39].

#### 4.2.2. MicroRNAs (miRNAs)

MicroRNAs (miRNAs) represent another important regulatory component in ovarian cancer. Changes in miRNA expression affect mRNA translation and modulate oncogenic signaling pathways. First discovered in nematodes in 1993 [38], miRNAs are 20–25 nucleotide non-coding RNAs present in eukaryotes, with regulatory functions. MicroRNAs can be isolated from a variety of biological sources, including tissues, cultured cells and bodily fluids such as blood, serum, plasma, urine and saliva. The fact that miRNAs are present in extracellular fluids, often in a stable form protected within vesicles or protein complexes, highlights their potential as non-invasive biomarkers [50].

MiRNAs regulate gene expression in organisms by inhibiting the translation of target mRNAs and degrading them. A single miRNA can regulate multiple mRNAs, thereby affecting the expression of multiple genes. Studies show that miRNAs play an important role in the development and progression of many diseases, including biological processes such as tumorigenesis, angiogenesis, and cell proliferation [110]. MiRNAs may be involved in cancer pathogenesis by acting as tumor suppressors, which has made them attractive for oncology study [111].

MicroRNAs (miRNAs) have been shown to influence virtually all cellular functions, including proliferation, apoptosis, and the cell cycle, and they are associated with many cancers. In epithelial ovarian cancer, the abnormal expression of several miRNAs may result in them acting as tumor suppressors or oncogenes [50] MiRNAs are secreted by specific cell types and can be found in extracellular vesicles in body fluids. Studies indicate that miRNAs are stable in body fluids, independent of intrinsic or extrinsic factors, and they undergo differential expression under different pathophysiological states [112]. Stable expression and high specificity in body fluids suggest that miRNA may serve as promising biomarkers for malignancies [50]. Selected miRNAs investigated in ovarian cancer and their potential diagnostic and prognostic relevance are summarized in Table 2. One example is miR-34a, an miRNA involved in cell cycle regulation, apoptosis and cellular stress response, whose reduced expression promotes tumor growth in ovarian cancer. MiR-34a is a direct target of the tumor suppressor TP53, and it is activated in response to DNA damage. In ovarian cancer, reduced miR-34a expression leads to uncontrolled tumor cell proliferation, inhibition of apoptosis, and increased resistance to chemotherapy. Consequently, this promotes tumor progression and poorer patient outcomes [113].

Various microRNAs (miRNAs), including miR-200, miR-506, miR-183 and miR-20, have been found to play a role in the development of ovarian cancer (OC) [40,48]. Several studies have also highlighted the prognostic significance of miR-3652 in ovarian cancer [113]. Furthermore, the literature suggests that high levels of miR-3652 are associated with poor survival outcomes and low levels are associated with higher survival outcomes [52]. Taken together, these results suggest that miR-3652 may serve as a prognostic biomarker, and the authors emphasize the urgent need for further research. The aim is to improve our understanding of the pathways modulated by microRNA to better employ them as diagnostic markers [116,117]. Furthermore, a special support vector regression (SVR)-based algorithm (OV-SURV) was employed that combines two data analysis methods. It combines support vector regression with a two-objective genetic algorithm for feature extraction enabling more accurate prediction of patient survival. Expression analysis showed that plasma and serum from ovarian cancer patients exhibit downregulation of miR-3652 [48,114], resulting in the dysregulation of tumor suppressor genes and the overexpression of tumor oncogenes. Among patients with ovarian cancer who showed a poor response to treatment, researchers found that restoring the expression of microRNA-3652 (miRNA-3652) may be associated with a reduction in chemoresistant cells. Treatment is typically achieved through therapies such as epigenetic drugs, which unblock previously silenced miRNAs [117,118]. Most of the available studies on miRNAs have generally focused on assessing their abundance and predicted role in causing ovarian cancer. However, none of these studies have yet presented a concise mechanism, demonstrating that miRNA-3652 causes ovarian cancer [48].

Other studies comparing microRNA (miRNA) expression profiles in malignant ovarian tumors and normal ovaries have identified hundreds of miRNAs with altered expression. Several independent studies have shown that miR-200 is one of these miRNAs, with increased expression in cancer cells [51,119]. The correlation between microRNA (miRNA) expression in urine and plasma was compared. Ultimately, they focused on three miRNAs: miR-200a, miR-200b, and miR-200c. The most significant finding was that all three miRNAs were detectable in both materials. Tumor and plasma analysis enabled malignant samples to be distinguished from benign ones. However, urine studies did not produce clear results. A correlation between miR-200 expression in urine and plasma was only observed in malignant samples. Therefore, while miRNAs show potential, further research is needed to clarify the usefulness of urine and plasma samples in monitoring ovarian cancer [49]. The expression patterns and clinical significance of non-coding RNAs can vary depending on the histological subtype and stage of ovarian cancer. While many microRNAs (miRNAs) and long non-coding RNAs (lncRNAs) have been identified as potential diagnostic or prognostic biomarkers, existing studies often focus on specific histological subtypes or include heterogeneous ovarian cancer patient populations. Therefore, the results of individual studies should be interpreted with caution and not generalized to all ovarian cancer subtypes and stages [53,120].

### 4.3. Adipocytokines as Non-Invasive Prognostic Biomarkers

In addition to endocrine disruptions, such as precocious puberty or late-onset menopause, obesity influences cancer development. Although less extensively studied, obesity has been associated with an increased risk for several cancers, including ovarian cancer [121]. Metabolic alterations, particularly glucose metabolism in both the body and the tumor microenvironment, have been linked to prognosis in ovarian cancer. Notably, glucose transporter expression is an important prognostic factor in OC [122].

In the UK, 512 out of 7011 ovarian cancer cases per year are thought to be associated with obesity [2,123]. Evidence also suggests an association between certain histological types of ovarian cancer, such as serous with low malignant potential and invasive mucinous tumors, with increased body mass index (BMI) [124] Huang et al. 2019 reported that BMI changes in young women aged 10–19 years may influence future ovarian cancer risk [125,126]. It is now known that adipose tissue is hormonally active and secretes numerous proteins with multidirectional effects, called adipokines. These are biologically active molecules produced mainly by adipocytes. Adipokines are involved not only in maintaining energy balance by influencing lipid metabolism, but also in maintaining adequate glucose levels and blood pressure. In addition, they also play an important role in maintaining endocrine balance [127,128,129]. Recent studies suggest that endocrine, paracrine and autocrine pathways have a significant impact on carcinogenesis, including breast, ovarian, and uterine cancers. Adipokines increase the risk of developing these cancers, particularly in postmenopausal women [130,131], supporting previous theories that adipose tissue and obesity have a significant impact on the incidence of cancer in women, particularly ovarian and endometrial malignancies [132,133]. Certain adipocytokines, such as adiponectin, leptin, chemerin and omentin, exhibit anti-cancer properties. These bioactive proteins secreted by adipose tissue influence metabolic and immunological processes [59,60,62]. Ovarian cancer patients with elevated serum leptin levels and overexpression of the leptin receptor (Ob-R) tend to present with more aggressive [61]. Leptin, a hormone produced mainly by adipocytes, is important in regulating satiety and controlling body weight. Physiologically, leptin regulates energy metabolism and interacts with hypothalamic centers to inhibit appetite and stimulate metabolism [134]. Leptin may also contribute to ovarian cancer development, as it is detectable in ovarian cancer cells, suggesting a role in promoting tumor cell growth and proliferation. Additionally, its angiogenic and mitogenic properties may support a role in tumorigenesis and elevated leptin levels in overweight individuals may increase the risk of ovarian cancer [135]. Studies on leptin receptor expression in ovarian cancer cell lines indicate its role in activating JAK2/STAT3 signaling and inhibiting apoptosis, highlighting its involvement in the regulation of ovarian cancer [2]. Additionally, adiponectin has been shown to stimulate the secretion of CXCL1 (C-X-C Motif Chemokine Ligand 1), a protein of the chemokine family that regulates the inflammatory response, cell activation and migration [136]. CXCL1 can promote angiogenesis independent of the Vascular Endothelial Growth Factor (VEGF), a key mediator in the formation of new blood vessels. Clinically, VEGF inhibitors are primarily used in the treatment of colorectal cancer and for intravitreal injections in ocular diseases [58]. Ouh et al. (2019) suggest that VEGF is a key initiator of angiogenesis in ovarian cancer. Given its role in modulating CXCL1-mediated angiogenesis, adiponectin may become a new therapeutic target for the treatment of this cancer, but its role in tumor growth and metastasis requires further investigation [57].

The tumor microenvironment plays a significant role in ovarian cancer progression and may contribute to heterogeneity in biomarker expression and clinical outcomes. Ovarian cancer cells interact with adipocytes, immune cells, endothelial cells, fibroblasts, and mesothelial cells through the exchange of cytokines, adipokines, growth factors, extracellular vesicles, and metabolic signals. Adipokines and inflammatory mediators present in ovarian cancer tissue, blood, and ascites fluid may influence tumor cell proliferation, migration, angiogenesis, and resistance to chemotherapy [61,129,135,136]. For instance, adiponectin stimulates the production of CXCL1 by ovarian cancer cells, promoting angiogenesis and tumor progression [57].

Malignant ascites fluid is an additional component of the ovarian cancer microenvironment that contains tumor cells, immune cells, cytokines, growth factors, extracellular vesicles, and metabolites. These factors interact with one another to promote tumor growth and spread to the peritoneal cavity. They also facilitate evasion of the immune response and resistance to treatment. Notably, adipokines and other soluble factors in ascites fluid may reflect the tumor microenvironment’s biological activity and have potential diagnostic or prognostic significance [129]. The composition of this microenvironment can vary among patients and change depending on disease stage and tumor characteristics. This variability may explain differences in biomarker expression and detectability among patients, so it should be considered when evaluating the efficacy of ovarian cancer biomarkers for diagnosis and prognosis.

### 4.4. Sodium-Dependent Phosphate Transport Protein 2B (NaPi2b) as a Therapeutic Target

**Figure 4:**
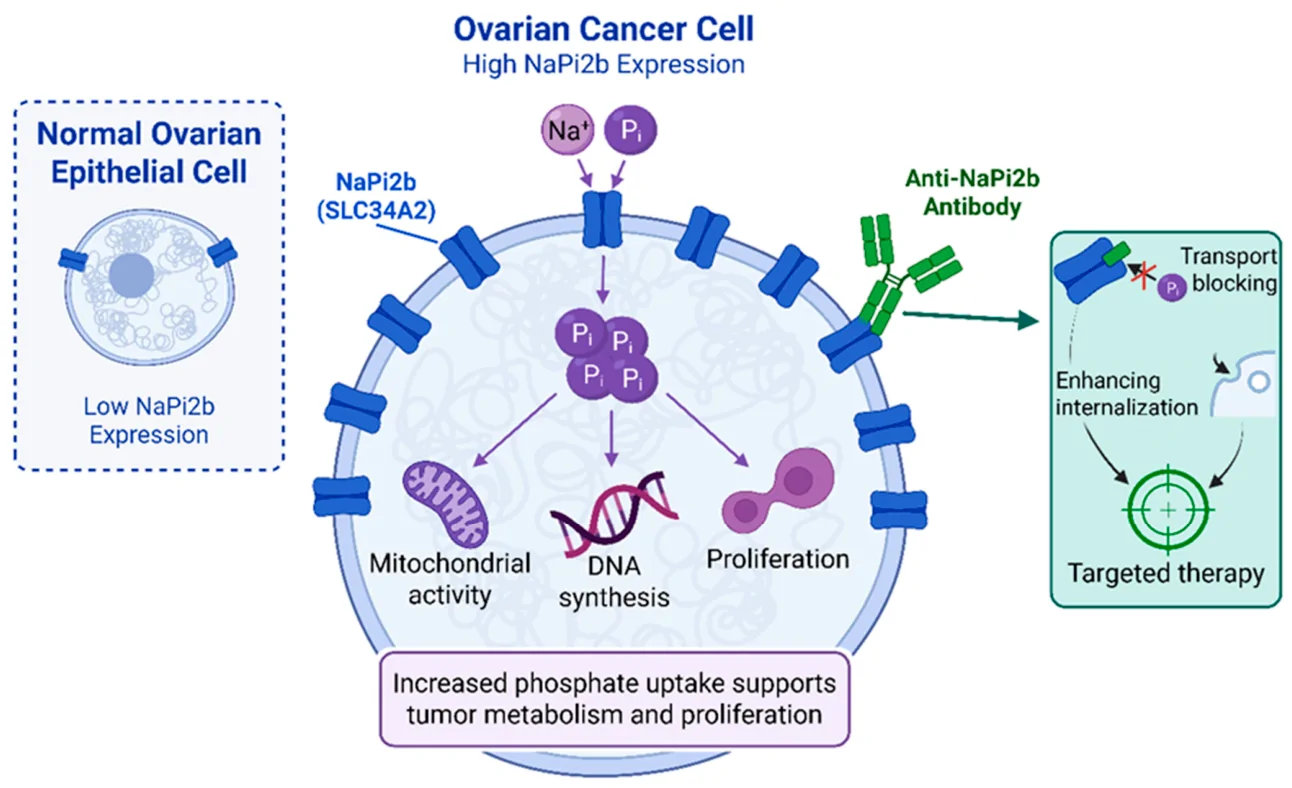
The role of sodium-dependent phosphate transport protein 2B (NaPi2B) and potential of Anti-NaPi2B antibodies in targeted therapy of ovarian cancer.

Another potential therapeutic target in ovarian cancer is sodium-dependent phosphate transport protein 2B (NaPi2b), a member of the SLC34 family of sodium-dependent phosphate transporters type 2. The SLC34 family includes NaPi2a (SLC34A1), NaPi2b and NaPi2c (SLC34A3) and plays a key role in phosphate homeostasis by mediating transport across epithelial membranes [60] (Figure 4). Membrane proteins account for at least 30% of all currently sequenced genomes and are targeted by 60% of approved drugs, making NaPi2B an attractive target for therapy. Furthermore, most anti-cancer agents target integral and membrane-bound proteins [126]. Na-Pi2b is overexpressed in multiple malignancies, including ovarian cancer [127], lung cancer [128], thyroid cancer [129] and colorectal cancer [130]. Although the main function in maintaining phosphate homeostasis in the human body is attributed to the best-studied NaPi2a, NaPi2b is involved in maintaining phosphate homeostasis through the uptake of unlimited phosphates in the small intestine and some organs and tissues [131]. Mouse antibodies against NaPi2b, such as the monoclonal antibodies L2(20/3) or MX35, have been shown to react strongly with the antigen on the surface of ovarian cancer cells, which may point to NaPi2b as an important therapeutic target in future ovarian cancer research [132]. A study [133], showed that the expression of the Solute Carrier Family 34 (Sodium Phosphate), Member 2 (SLC34A2) gene, which encodes the NaPi2b protein, may serve as a marker to monitor or predict response to neoadjuvant targeted therapy in patients with ovarian cancer. In addition, Levan et al., 2017 [134], highlights the growing interest in the development of NaPi2b-targeting monoclonal antibody-based therapies for ovarian cancer. However, they also emphasize the need for further knowledge and characterization of the expression pattern of this protein. Monoclonal antibodies can also be used in antibody–drug conjugate (ADC) therapies. Preclinical studies in mice have shown that monomethylauristatin E (MMAE), an ADC targeting NaPi2b, is an effective new therapy for the treatment of ovarian cancer and non-small cell lung cancer (NSCLC). MMAE-conjugated anti-NaPi2b ADC has specific anti-proliferative activity in tumor cells in vitro and anti-tumor activity in NaPi2b-expressing cancer xenograft models. Conjugation of MMAE with an anti-NaPi2b monoclonal antibody improved tolerability and extended the therapeutic window of MMAE in animal studies. Additional cytotoxicity studies of this compound in monkeys showed acceptable safety levels. In conclusion, the anti-NaPi2b ADC demonstrated significant efficacy in non-clinical NaPi2b-expressing models and acceptable safety in animal studies suggesting that anti-NaPi2b ADC may be a promising drug for the treatment of ovarian cancer. Based on promising results in preclinical studies, anti-NaPi2b ADCs have entered clinical development for both ovarian and lung cancer [135,136].

However, the therapeutic potential of NaPi2b should be considered in the context of its expression in healthy tissues. NaPi2b is not exclusively found in cancer cells. In fact, it is physiologically expressed in many epithelial tissues. Studies have shown that NaPi2b is present in the lungs, kidneys, intestines, and mammary glands. However, the level and distribution of expression varies by tissue [137]. Immunohistochemical analyses have demonstrated NaPi2b expression in normal epithelial tissues, including the lungs and mammary glands. However, particularly high expression was detected in the human kidney [137]. Therefore, when developing NaPi2b-targeted therapies, especially antibody–drug conjugates, NaPi2b expression should be considered a significant factor. Targeted delivery of a cytotoxic payload may result in toxicity in tissues outside the tumor. The high prevalence of NaPi2b expression in ovarian cancer may not be sufficient to establish its therapeutic value. Thus, comparing NaPi2b expression in tumor and healthy tissues is essential for evaluating tumor selectivity and the therapeutic potential of NaPi2b-targeted therapies.

## 5. Summary

Despite significant medical advances, ovarian cancer remains one of the deadliest gynecological cancers worldwide. One of the main reasons for this is the lack of effective early detection methods, which consequently leads to a high mortality rate. The current knowledge regarding established and newly developed ovarian cancer biomarkers, as well as their potential prognostic and therapeutic applications, has been thoroughly considered. Additionally, researchers are exploring avenues for future studies aimed at improving treatment outcomes for this disease. Current data show that, despite its key role in monitoring treatment and detecting disease recurrence, the CA125 marker has limited value in early diagnosis due to its elevated levels in milder conditions. HE4 exhibits greater specificity than CA125, particularly in distinguishing between benign and malignant lesions. Its combination with CA125, along with the use of the ROMA risk assessment algorithm, improves diagnostic effectiveness, though this depends on patients’ clinical characteristics. Furthermore, there is increasing interest in biomarkers derived from exosomes, microRNAs (miRNAs), and cell-free tumor DNA (ctDNA), as they may enable earlier detection of ovarian cancer. Adipokines, on the other hand, are ideal for prognosis rather than diagnosis. NaPi2b, on the other hand, is a promising therapeutic target for antibody–drug conjugates. However, its safety in clinical settings requires further study.

### 5.1. Limitations

Despite the significant need for and extensive research on new biomarkers for ovarian cancer, their clinical application remains limited. Several factors contribute to this limitation. One of the most significant problems is the absence of a single marker with sufficient sensitivity and specificity to effectively detect the disease in its early stages. Commonly used biomarkers, such as CA125 and HE4, have significant limitations despite their proven diagnostic and prognostic efficacy. CA125 levels may rise in benign conditions as well as inflammatory conditions and endometriosis. HE4 is not effective in all subtypes of ovarian cancer, despite its higher specificity.

Another limitation is the need to use cancer screening algorithms such as ROMA, which hinders standardization of diagnosis. Additionally, biomarker levels are subject to interindividual variability and external factors. Patient-related factors such as age, weight, lifestyle, and comorbidities can influence these levels, increasing the likelihood of false positives and false negatives. Another limitation is the need to use screening algorithms such as ROMA. Using these algorithms may hinder the standardization and unambiguous assessment of different patient populations. Biomarker levels are associated with various patient-specific factors, including age, body weight, lifestyle, hormonal status, and comorbidities. These factors can lead to false-positive or false-negative results.

Modern diagnostic technologies, including the analysis of circulating tumor DNA, microRNAs (miRNAs), and proteomics, also pose a significant challenge. Despite their enormous potential, these technologies have significant limitations related to high costs, complex testing procedures, and limited availability in current clinical practice.

Another significant limitation is new biomarkers, such as adipocytokines. Preliminary results highlight their potential diagnostic and prognostic significance. Unfortunately, the lack of conclusive data and insufficient clinical trials currently limit their applicability. Similar limitations apply to new therapeutic targets such as NaPi2b. Although therapies targeting this transporter exist have shown promising results, their efficacy and safety must be confirmed in larger clinical trials.

The current limitations associated with the use of ovarian cancer biomarkers primarily stem from the insufficient validation of their clinical efficacy and the need to determine the impact of individual patient factors. Despite the promising data on these markers, their implementation into routine diagnostics requires further research and standardization.

### 5.2. Future Directions

Despite significant advances in diagnosis, ovarian cancer remains a serious clinical challenge due to late diagnosis and the lack of reliable early detection methods. Therefore, identifying new targeted therapies and biomarkers and improving our understanding of the tumor microenvironment remain essential.

In the context of microRNAs (miRNAs), future research should focus on analyzing them using liquid biopsies, which could significantly improve disease detection and monitoring. Additionally, analyzing circulating tumor DNA (ctDNA) is a potentially more effective method for detecting mutations and disease progression.

Future research on adipocytokines should investigate their precise impact on the tumor microenvironment. Given that adiponectin stimulates angiogenesis and processes independent of VEGF, it appears to play a significant role in developing pathological tumor vasculature. Understanding these mechanisms will help us determine the role of adipocytokines in ovarian cancer pathogenesis and assess their potential as therapeutic targets. Future therapies targeting NaPi2b should focus on its precise expression patterns in different types of ovarian cancer. Additionally, patient groups that would benefit most from these therapies should be identified. A key issue is assessing the safety and efficacy of antibody–drug conjugates (ADCs) in clinical trials. It is crucial to determine their potential advantage over standard treatment methods. A more detailed understanding of the mechanisms responsible for responses to anti-NaPi2b therapy could lead to the development of more precise therapeutic strategies and the tailoring of treatment to patients’ individual needs.

Current data suggest that future research will focus on multi-marker strategies combining molecular biomarkers, risk assessment algorithms and advanced analytical technologies. The most important goal remains identifying markers that can reliably detect ovarian cancer at an early stage, which could significantly reduce mortality among patients.

## Figures and Tables

**Table 2:** Selected miRNAs in ovarian cancer.

Marker	Sample Type	Role in Diagnosis/Prognosis	References
**miR-200**	ovarian tumor tissue	Its increased expression is frequently observed in cancer cells, which can help to distinguish between malignant and benign lesions.	[40,49]
**miR-506**	ovarian tumor tissue (frozen and FFPE/TMA	Its expression is associated with inhibiting the proliferation of cancer cells and inducing apoptosis.	[114]
**miR-183**	ovarian tumor tissue	It promotes the proliferation of cancer cells and tumor progression, and its level may indicate how aggressive ovarian cancer is.	[115]
**miR-20**	ovarian tumor tissue	It promotes the development of OC by encouraging the proliferation of cancer cells and inhibiting apoptosis. Its level can also serve as an indicator of prognosis during the course of the disease.	[48]
**miR-34a**	MicroRNA	Regulates apoptosis and proliferation; it is a potential tumor suppressor.	[51]
**miR-1271-5p**	MicroRNA	As a tumor suppressor, it inhibits cell proliferation, migration and tumor invasiveness.	[39]

## Data Availability

No new data were created or analyzed in this study. Data sharing is not applicable to this article.

## References

[1] Zhang R., Siu M.K.Y., Ngan H.Y.S., Chan K.K.L. (2022). Molecular Biomarkers for the Early Detection of Ovarian Cancer. Int. J. Mol. Sci..

[2] Kukla A., Piotrowska K., Misiek M., Chudecka-Glaz A.M. (2022). Role of Adipokines in Ovarian Cancer Epidemiology and Prognosis. Ginekol. Pol..

[3] Xiao Y., Bi M., Guo H., Li M. (2022). Multi-Omics Approaches for Biomarker Discovery in Early Ovarian Cancer Diagnosis. eBioMedicine.

[4] Sung H., Ferlay J., Siegel R.L., Laversanne M., Soerjomataram I., Jemal A., Bray F. (2021). Global Cancer Statistics 2020: GLOBOCAN Estimates of Incidence and Mortality Worldwide for 36 Cancers in 185 Countries. CA Cancer J. Clin..

[5] Wang G., Yang H., Wang Y., Qin J. (2025). Ovarian Cancer Targeted Therapy: Current Landscape and Future Challenges. Front. Oncol..

[6] Jung E.M., Moran V.O., Engel M., Krüger-Genge A., Stroszczynski C., Jung F. (2023). Modified Contrast-Enhanced Ultrasonography with the New High-Resolution Examination Technique of High Frame Rate Contrast-Enhanced Ultrasound (HiFR-CEUS) for Characterization of Liver Lesions: First Results. Clin. Hemorheol. Microcirc..

[7] Ettorre V.M., AlAshqar A., Sethi N., Santin A.D. (2025). Personalized Treatment in Ovarian Cancer: A Review of Disease Monitoring, Biomarker Expression, and Targeted Treatments for Advanced, Recurrent Ovarian Cancers. Cancers.

[8] Jamali Z., Razipour M., Zargar M., Ghasemnejad-Berenji H., Akrami S.M. (2025). Ovarian Cancer Extracellular Vesicle Biomarkers. Clin. Chim. Acta.

[9] Felder M., Kapur A., Gonzalez-Bosquet J., Horibata S., Heintz J., Albrecht R., Fass L., Kaur J., Hu K., Shojaei H. (2014). MUC16 (CA125): Tumor Biomarker to Cancer Therapy, a Work in Progress. Mol. Cancer.

[10] Bălăceanu L.A., Grigore C., Dina I., Gurău C.D., Mihai M.M., Bălăceanu-Gurău B. (2025). CA125 as a Potential Biomarker in Non-Malignant Serous Effusions: Diagnostic and Prognostic Considerations. J. Clin. Med..

[11] Zhang M., Cheng S., Jin Y., Zhao Y., Wang Y. (2021). Roles of CA125 in Diagnosis, Prediction, and Oncogenesis of Ovarian Cancer. Biochim. Biophys. Acta Rev. Cancer.

[12] Cancer Tomorrow. https://gco.iarc.fr/tomorrow/en/dataviz/tables.

[13] Zhu B., Gu H., Mao Z., Beeraka N.M., Zhao X., Anand M.P., Zheng Y., Zhao R., Li S., Manogaran P. (2024). Global Burden of Gynaecological Cancers in 2022 and Projections to 2050. J. Glob. Health.

[14] Moffitt L., Karimnia N., Stephens A., Bilandzic M. (2019). Therapeutic Targeting of Collective Invasion in Ovarian Cancer. Int. J. Mol. Sci..

[15] Hong M.K., Ding D.C. (2025). Early Diagnosis of Ovarian Cancer: A Comprehensive Review of the Advances, Challenges, and Future Directions. Diagnostics.

[16] Thomakos N., Diakosavvas M., Machairiotis N., Fasoulakis Z., Zarogoulidis P., Rodolakis A. (2019). Rare Distant Metastatic Disease of Ovarian and Peritoneal Carcinomatosis: A Review of the Literature. Cancers.

[17] De Oliveira L.R.L.B., Horvat N., Andrieu P.I.C., Panizza P.S.B., Cerri G.G., Viana P.C.C. (2021). Ovarian Cancer Staging: What the Surgeon Needs to Know. Br. J. Radiol..

[18] Elias K.M., Guo J., Bast R.C. (2018). Early Detection of Ovarian Cancer. Hematol. Oncol. Clin. N. Am..

[19] Yamagami W., Nagase S., Takahashi F., Ino K., Hachisuga T., Mikami M., Enomoto T., Katabuchi H., Aoki D. (2020). A Retrospective Study for Investigating the Relationship between Old and New Staging Systems with Prognosis in Ovarian Cancer Using Gynecologic Cancer Registry of Japan Society of Obstetrics and Gynecology (JSOG): Disparity between Serous Carcinoma and Clear cell carcinoma. J. Gynecol. Oncol..

[20] Mustafin C., Vesnin S., Turnbull A., Dixon M., Goltsov A., Goryanin I. (2022). Diagnostics of Ovarian Tumors in Postmenopausal Patients. Diagnostics.

[21] Rosenthal A.N., Fraser L.S.M., Philpott S., Manchanda R., Burnell M., Badman P., Hadwin R., Rizzuto I., Benjamin E., Singh N. (2017). Evidence of Stage Shift in Women Diagnosed with Ovarian Cancer During Phase II of the United Kingdom Familial Ovarian Cancer Screening Study. J. Clin. Oncol..

[22] Charkhchi P., Cybulski C., Gronwald J., Wong F.O., Narod S.A., Akbari M.R. (2020). CA125 and Ovarian Cancer: A Comprehensive Review. Cancers.

[23] Ndukwe M.I., Pavone M., Habes D., Bizzarri N., Stepan M., Bretová P., Mascilini F., Karasek D., Pohankova D., Ubom A. (2025). The Role of Ultrasound-Guided Tru-Cut Biopsy in Ovarian Cancer: A Systematic Review of Its Safety, Adequacy, and Accuracy with Meta-Analysis of Diagnostic Performance. J. Ovarian Res..

[24] Bonifácio V.D.B. (2020). Ovarian Cancer Biomarkers: Moving Forward in Early Detection. Tumor Microenvironment.

[25] Campbell S., Gentry-Maharaj A. (2018). The Role of Transvaginal Ultrasound in Screening for Ovarian Cancer. Climacteric.

[26] Jurgiel W.A., Panasiuk B., Posmyk R. (2026). Hereditary Ovarian Cancer. Discov. Oncol..

[27] Popat V., Han E. (2025). From Fallopian Tube to Ovarian Cancer: Understanding the Evaluation and Management of Serous Tubal Intraepithelial Carcinoma Lesions. Curr. Treat. Options Oncol..

[28] Ansenberger K., Zhuge Y., Lagman J.A.J., Richards C., Barua A., Bahr J.M., Hales D.B. (2009). E-Cadherin Expression in Ovarian Cancer in the Laying Hen, Gallus Domesticus, Compared to Human Ovarian Cancer. Gynecol. Oncol..

[29] Choi P.W., So W.W., Yang J., Liu S., Tong K.K., Kwan K.M., Kwok J.S.L., Tsui S.K.W., Ng S.K., Hales K.H. (2020). MicroRNA-200 Family Governs Ovarian Inclusion Cyst Formation and Mode of Ovarian Cancer Spread. Oncogene.

[30] Chacko N., Ankri R. (2024). Non-Invasive Early-Stage Cancer Detection: Current Methods and Future Perspectives. Clin. Exp. Med..

[31] Ghose A., McCann L., Makker S., Mukherjee U., Gullapalli S.V.N., Erekkath J., Shih S., Mahajan I., Sanchez E., Uccello M. (2024). Diagnostic Biomarkers in Ovarian Cancer: Advances beyond CA125 and HE4. Ther. Adv. Med. Oncol..

[32] Taliento C., Morciano G., Nero C., Froyman W., Vizzielli G., Pavone M., Salvioli S., Tormen M., Fiorica F., Scutiero G. (2024). Circulating Tumor DNA as a Biomarker for Predicting Progression-Free Survival and Overall Survival in Patients with Epithelial Ovarian Cancer: A Systematic Review and Meta-Analysis. Int. J. Gynecol. Cancer.

[33] Drapkin R., Von Horsten H.H., Lin Y., Mok S.C., Crum C.P., Welch W.R., Hecht J.L. (2005). Human Epididymis Protein 4 (HE4) Is a Secreted Glycoprotein That Is Overexpressed by Serous and Endometrioid Ovarian Carcinomas. Cancer Res..

[34] Angeloni A., De Vito C., Farina A., Terracciano D., Cennamo M., Passerini R., Bottari F., Schirinzi A., Vettori R., Steffan A. (2022). New Analytical Approach for the Alignment of Different HE4 Automated Immunometric Systems: An Italian Multicentric Study. J. Clin. Med..

[35] Anastasi E., Farina A., Granato T., Colaiacovo F., Pucci B., Tartaglione S., Angeloni A. (2023). Recent Insight about HE4 Role in Ovarian Cancer Oncogenesis. Int. J. Mol. Sci..

[36] Gryshkova V., Goncharuk I., Gurtovyy V., Khozhayenko Y., Nespryadko S., Vorobjova L., Usenko V., Gout I., Filonenko V., Kiyamova R. (2009). The Study of Phosphate Transporter NAPI2B Expression in Different Histological Types of Epithelial Ovarian Cancer. Exp. Oncol..

[37] Bulatova L., Savenkova D., Nurgalieva A., Reshetnikova D., Timonina A., Skripova V., Bogdanov M., Kiyamova R. (2022). Toward a Topology-Based Therapeutic Design of Membrane Proteins: Validation of NaPi2b Topology in Live Ovarian Cancer Cells. Front. Mol. Biosci..

[38] Lee R.C., Feinbaum R.L., Ambros V. (1993). The *C. elegans* Heterochronic Gene Lin-4 Encodes Small RNAs with Antisense Complementarity to Lin-14. Cell.

[39] Hou J., Zhang G., Wang X., Wang Y., Wang K. (2023). Functions and Mechanisms of LncRNA MALAT1 in Cancer Chemotherapy Resistance. Biomark. Res..

[40] Hu X., Macdonald D.M., Huettner P.C., Feng Z., El Naqa I.M., Schwarz J.K., Mutch D.G., Grigsby P.W., Powell S.N., Wang X. (2009). A MiR-200 MicroRNA Cluster as Prognostic Marker in Advanced Ovarian Cancer. Gynecol. Oncol..

[41] Sahin K., Yenice E., Tuzcu M., Orhan C., Mizrak C., Ozercan I.H., Sahin N., Yilmaz B., Bilir B., Ozpolat B. (2018). Lycopene Protects Against Spontaneous Ovarian Cancer Formation in Laying Hens. J. Cancer Prev..

[42] Dikshit A., Hales K., Hales D.B. (2017). Whole Flaxseed Diet Alters Estrogen Metabolism to Promote 2-Methoxtestradiol-Induced Apoptosis in Hen Ovarian Cancer. J. Nutr. Biochem..

[43] King S.M., Burdette J.E. (2011). Evaluating the Progenitor Cells of Ovarian Cancer: Analysis of Current Animal Models. BMB Rep..

[44] Niebora J., Data K., Domagała D., Józkowiak M., Barrett S., Norizadeh Abbariki T., Bryja A., Kulus M., Woźniak S., Ziemak H. (2024). Avian Models for Human Carcinogenesis-Recent Findings from Molecular and Clinical Research. Cells.

[45] Pal P., Starkweather K.N., Hales K.H., Hales D.B. (2021). A Review of Principal Studies on the Development and Treatment of Epithelial Ovarian Cancer in the Laying Hen Gallus Gallus. Comp. Med..

[46] Ciucci A., Buttarelli M., Fagotti A., Scambia G., Gallo D. (2022). Preclinical Models of Epithelial Ovarian Cancer: Practical Considerations and Challenges for a Meaningful Application. Cell. Mol. Life Sci..

[47] Tudrej P., Kujawa K.A., Cortez A.J., Lisowska K.M. (2019). Characteristics of In Vivo Model Systems for Ovarian Cancer Studies. Diagnostics.

[48] Imran K., Iqbal M.J., Abid R., Ahmad M.M., Calina D., Sharifi-Rad J., Cho W.C. (2023). Cellular Signaling Modulated by MiRNA-3652 in Ovarian Cancer: Unveiling Mechanistic Pathways for Future Therapeutic Strategies. Cell Commun. Signal..

[49] Savolainen K., Savolainen K., Scaravilli M., Scaravilli M., Scaravilli M., Ilvesmäki A., Staff S., Staff S., Staff S., Tolonen T. (2020). Expression of the MiR-200 Family in Tumor Tissue, Plasma and Urine of Epithelial Ovarian Cancer Patients in Comparison to Benign Counterparts. BMC Res. Notes.

[50] Zhao L., Liang X., Wang L., Zhang X. (2022). The Role of MiRNA in Ovarian Cancer: An Overview. Reprod. Sci..

[51] Liu F., An Z., Tang C., Han X. (2025). Potential Function of MiRNA as a Diagnostic Biomarker for Human Ovarian Cancer. Discov. Oncol..

[52] Sathipati S.Y., Ho S.Y. (2021). Identification of the MiRNA Signature Associated with Survival in Patients with Ovarian Cancer. Aging.

[53] Calura E., Fruscio R., Paracchini L., Bignotti E., Ravaggi A., Martini P., Sales G., Beltrame L., Clivio L., Ceppi L. (2013). MiRNA Landscape in Stage I Epithelial Ovarian Cancer Defines the Histotype Specificities. Clin. Cancer Res..

[54] Sharbatoghli M., Vafaei S., Aboulkheyr Es H., Asadi-Lari M., Totonchi M., Madjd Z. (2020). Prediction of the Treatment Response in Ovarian Cancer: A CtDNA Approach. J. Ovarian Res..

[55] Yan X., Su J., Wang Z. (2025). Revolution of Circulating Tumor DNA: From Bench Innovations to Bedside Implementations. Curr. Issues Mol. Biol..

[56] Yang W.L., Gentry-Maharaj A., Simmons A., Ryan A., Fourkala E.O., Lu Z., Baggerly K.A., Zhao Y., Lu K.H., Bowtell D. (2017). Elevation of TP53 Autoantibody Before CA125 in Preclinical Invasive Epithelial Ovarian Cancer. Clin. Cancer Res..

[57] Ouh Y.T., Cho H.W., Lee J.K., Choi S.H., Choi H.J., Hong J.H. (2019). CXC Chemokine Ligand 1 Mediates Adiponectin-Induced Angiogenesis in Ovarian Cancer. Tumor Biol..

[58] Kim L.A., D’Amore P.A. (2012). A Brief History of Anti-VEGF for the Treatment of Ocular Angiogenesis. Am. J. Pathol..

[59] Booth A., Magnuson A., Fouts J., Foster M. (2015). Adipose Tissue, Obesity and Adipokines: Role in Cancer Promotion. Horm. Mol. Biol. Clin. Investig..

[60] Funcke J.B., Scherer P.E. (2019). Beyond Adiponectin and Leptin: Adipose Tissue-Derived Mediators of Inter-Organ Communication. J. Lipid Res..

[61] Ray A., Fornsaglio J., Dogan S., Hedau S., Naik S.D., De A. (2018). Gynaecological Cancers and Leptin: A Focus on the Endometrium and Ovary. Facts Views Vis. ObGyn.

[62] Dec P., Poniewierska-Baran A., Modrzejewski A., Pawlik A. (2023). The Role of Omentin-1 in Cancers Development and Progression. Cancers.

[63] Høgdall E.V.S., Christensen L., Kjaer S.K., Blaakaer J., Kjærbye-Thygesen A., Gayther S., Jacobs I.J., Høgdall C.K. (2007). CA125 Expression Pattern, Prognosis and Correlation with Serum CA125 in Ovarian Tumor Patients. From The Danish “MALOVA” Ovarian Cancer Study. Gynecol. Oncol..

[64] Sturgeon C.M., Duffy M.J., Walker G. (2011). The National Institute for Health and Clinical Excellence (NICE) Guidelines for Early Detection of Ovarian Cancer: The Pivotal Role of the Clinical Laboratory. Ann. Clin. Biochem..

[65] Funston G., Mounce L.T.A., Price S., Rous B., Crosbie E.J., Hamilton W., Walter F.M. (2021). CA125 Test Result, Test-to-Diagnosis Interval, and Stage in Ovarian Cancer at Diagnosis: A Retrospective Cohort Study Using Electronic Health Records. Br. J. Gen. Pract..

[66] Mukama T., Fortner R.T., Katzke V., Hynes L.C., Petrera A., Hauck S.M., Johnson T., Schulze M., Schiborn C., Rostgaard-Hansen A.L. (2022). Prospective Evaluation of 92 Serum Protein Biomarkers for Early Detection of Ovarian Cancer. Br. J. Cancer.

[67] Gu Z., He Y., Zhang Y., Chen M., Song K., Huang Y., Li Q., Di W. (2018). Postprandial Increase in Serum CA125 as a Surrogate Biomarker for Early Diagnosis of Ovarian Cancer. J. Transl. Med..

[68] Forster I.C., Hernando N., Biber J., Murer H. (2013). Phosphate Transporters of the SLC20 and SLC34 Families. Mol. Asp. Med..

[69] Wilczyński J., Paradowska E., Wilczyński M. (2024). High-Grade Serous Ovarian Cancer—A Risk Factor Puzzle and Screening Fugitive. Biomedicines.

[70] Cramer D.W., Bast R.C., Berg C.D., Diamandis E.P., Godwin A.K., Hartge P., Lokshin A.E., Lu K.H., McIntosh M.W., Mor G. (2011). Ovarian Cancer Biomarker Performance in Prostate, Lung, Colorectal, and Ovarian Cancer Screening Trial Specimens. Cancer Prev. Res..

[71] Giuseppe M., Francesco P., Corrado T., Roberto M., Carlo D.C.N., Federica G., Daniela L., Alessandra G., Teresa S., Gianmarco R. (2020). Prognostic Role of Human Epididymis Protein _4_ (HE_4_) in Ovarian Cancer Treatment: Our Point of View. Arch. Cancer Biol. Ther..

[72] Wei S., Li H., Zhang B. (2016). The Diagnostic Value of Serum HE4 and CA-125 and ROMA Index in Ovarian Cancer. Biomed. Rep..

[73] Granato T., Porpora M.G., Longo F., Angeloni A., Manganaro L., Anastasi E. (2015). HE4 in the Differential Diagnosis of Ovarian Masses. Clin. Chim. Acta.

[74] Hamed E.O., Ahmed H., Sedeek O.B., Mohammed A.M., Abd-Alla A.A., Ghaffar H.M.A. (2013). Significance of HE4 Estimation in Comparison with CA125 in Diagnosis of Ovarian Cancer and Assessment of Treatment Response. Diagn. Pathol..

[75] Cviič D., Jagarlamudi K., Meglič L., Škof E., Zore A., Lukanović D., Eriksson S., Osredkar J. (2023). A Dual Biomarker TK1 Protein and CA125 or HE4-Based Algorithm as a Better Diagnostic Tool than ROMA Index in Early Detection of Ovarian Cancer. Cancers.

[76] Samborski A., Miller M.C., Blackman A., Maclaughlan-David S., Jackson A., Lambert-Messerlian G., Rowswell-Turner R., Moore R.G. (2022). HE4 and CA125 Serum Biomarker Monitoring in Women with Epithelial Ovarian Cancer. Tumor Biol..

[77] Köbel M., Kalloger S.E., Boyd N., McKinney S., Mehl E., Palmer C., Leung S., Bowen N.J., Ionescu D.N., Rajput A. (2008). Ovarian Carcinoma Subtypes Are Different Diseases: Implications for Biomarker Studies. PLoS Med..

[78] Blackman A., Mitchell J., Rowswell-Turner R., Singh R., Kim K.K., Eklund E., Skates S., Bast R.C., Messerlian G., Miller M.C. (2021). Analysis of Serum HE4 Levels in Various Histologic Subtypes of Epithelial Ovarian Cancer and Other Malignant Tumors. Tumor Biol..

[79] Wang J., Gao J., Yao H., Wu Z., Wang M., Qi J. (2014). Diagnostic Accuracy of Serum HE4, CA125 and ROMA in Patients with Ovarian Cancer: A Meta-Analysis. Tumor Biol..

[80] Olsen M., Lof P., Stiekema A., van den Broek D., Wilthagen E.A., Bossuyt P.M., Lok C.A.R. (2021). The Diagnostic Accuracy of Human Epididymis Protein 4 (HE4) for Discriminating between Benign and Malignant Pelvic Masses: A Systematic Review and Meta-Analysis. Acta Obstet. Gynecol. Scand..

[81] Biatta C.M., Paudice M., Greppi M., Parrella V., Parodi A., De Luca G., Cerruti G.M., Mammoliti S., Caroti C., Menichini P. (2023). The Fading Guardian: Clinical Relevance of TP53 Null Mutation in High-Grade Serous Ovarian Cancers. Front. Immunol..

[82] Montero-Calle A., Garranzo-Asensio M., Moreno-Casbas M.T., Campuzano S., Barderas R. (2024). Autoantibodies in Cancer: A Systematic Review of Their Clinical Role in the Most Prevalent Cancers. Front. Immunol..

[83] Fiala C., Kulasingam V., Diamandis E.P. (2018). Circulating Tumor DNA for Early Cancer Detection. J. Appl. Lab. Med..

[84] Xue V.W., Wong C.S.C., Cho W.C.S. (2019). Early Detection and Monitoring of Cancer in Liquid Biopsy: Advances and Challenges. Expert Rev. Mol. Diagn..

[85] Parums D.V. (2025). A Review of Circulating Tumor DNA (CtDNA) and the Liquid Biopsy in Cancer Diagnosis, Screening, and Monitoring Treatment Response. Med. Sci. Monit..

[86] Kumar M., Choudhury Y., Ghosh S.K., Mondal R. (2018). Application and Optimization of Minimally Invasive Cell-Free DNA Techniques in Oncogenomics. Tumor Biol..

[87] Reece M., Saluja H., Hollington P., Karapetis C.S., Vatandoust S., Young G.P., Symonds E.L. (2019). The Use of Circulating Tumor DNA to Monitor and Predict Response to Treatment in Colorectal Cancer. Front. Genet..

[88] Yao G., Zhang Y., Li H., Teng L., Man Y., Wang S., Liu C., Fan Y., Chen D. (2026). Circulating Tumor DNA as a Biomarker in Pancreatic Cancer: Clinical Applications and Challenges. Clin. Transl. Gastroenterol..

[89] Pepe L., Zuccalà V., Giordano W.G., Giuffrè G., Martini M., Cianci V., Mondello C., Berretta M., Cianci S., Fiorentino V. (2026). Circulating Tumor DNA in Ovarian Cancer: Emerging Roles in Early Detection, Risk Stratification, and Disease Monitoring. Cancers.

[90] Pascual J., Attard G., Bidard F.C., Curigliano G., De Mattos-Arruda L., Diehn M., Italiano A., Lindberg J., Merker J.D., Montagut C. (2022). ESMO Recommendations on the Use of Circulating Tumour DNA Assays for Patients with Cancer: A Report from the ESMO Precision Medicine Working Group. Ann. Oncol..

[91] Herberts C., Wyatt A.W. (2021). Technical and Biological Constraints on CtDNA-Based Genotyping. Trends Cancer.

[92] Abulajiang Y., Ming W., Wu Y. (2026). The Role of Circulating Tumor DNA in Ovarian Cancer. Discov. Oncol..

[93] Semenkovich N.P., Szymanski J.J., Earland N., Chauhan P.S., Pellini B., Chaudhuri A.A. (2023). Genomic Approaches to Cancer and Minimal Residual Disease Detection Using Circulating Tumor DNA. J. Immunother. Cancer.

[94] Li X., Liu Y., Zheng S., Zhang T., Wu J., Sun Y., Zhang J., Liu G. (2021). Role of Exosomes in the Immune Microenvironment of Ovarian Cancer. Oncol. Lett..

[95] Chen B., Qiu X., Li Y. (2025). Exosomes in Ovarian Cancer: Impact on Drug Resistance and Advances in SERS Detection Techniques. J. Pharm. Anal..

[96] Niebora J., Woźniak S., Domagała D., Data K., Farzaneh M., Zehtabi M., Dari M.A.G., Pour F.K., Bryja A., Kulus M. (2024). The Role of NcRNAs and Exosomes in the Development and Progression of Endometrial Cancer. Front. Oncol..

[97] Hamzah R.N., Alghazali K.M., Biris A.S., Griffin R.J. (2021). Exosome Traceability and Cell Source Dependence on Composition and Cell-Cell Cross Talk. Int. J. Mol. Sci..

[98] Othman N., Jamal R., Abu N. (2019). Cancer-Derived Exosomes as Effectors of Key Inflammation-Related Players. Front. Immunol..

[99] Pan J., Ding M., Xu K., Yang C., Mao L.J. (2017). Exosomes in Diagnosis and Therapy of Prostate Cancer. Oncotarget.

[100] Wu M., Wang G., Hu W., Yao Y., Yu X.F. (2019). Emerging Roles and Therapeutic Value of Exosomes in Cancer Metastasis. Mol. Cancer.

[101] Vanni I., Alama A., Grossi F., Dal Bello M.G., Coco S. (2017). Exosomes: A New Horizon in Lung Cancer. Drug Discov. Today.

[102] Jorgensen A., Vermeer D.W., Jung E., Williamson C.S., Omran D.K., Schwartz L.E., Yoon J.Y., Barr J., Tetlow A.L., Trinidad C.V. (2026). Tumor-Infiltrating Nociceptor Neurons in Ovarian Cancer Treatment Resistance. Adv. Biol..

[103] Ou L., Feng C., Sun C., Xue Z., Liu D., Gong Q., An Y., Luo Y., Sheng P., Wang Z. (2026). Neural Remodeling Shapes a Nerve-Dependent Metastatic Ecosystem in Ovarian Cancer. Pharmacol. Res..

[104] Yokoi A., Yoshioka Y., Yamamoto Y., Ishikawa M., Ikeda S.I., Kato T., Kiyono T., Takeshita F., Kajiyama H., Kikkawa F. (2017). Malignant Extracellular Vesicles Carrying MMP1 MRNA Facilitate Peritoneal Dissemination in Ovarian Cancer. Nat. Commun..

[105] Wang J., Yue B.L., Huang Y.Z., Lan X.Y., Liu W.J., Chen H. (2022). Exosomal RNAs: Novel Potential Biomarkers for Diseases—A Review. Int. J. Mol. Sci..

[106] Wagner V., Morton M., Dorayappan K.D.P., Gonzalez A., Yu L., Sakaue T., Conrads T., Maxwell G.L., Cosgrove C., Backes F. (2025). Circulating Extracellular Vesicles Protein Expression for Early Prediction of Platinum-Resistance in High-Grade Serous Ovarian Cancer. Oncogene.

[107] Liu Y., Liu H., Zhu C., Yang Y., Shen Z., Shan G., Chen L., Zhou Y. (2025). Tumor Small Extracellular Vesicle-Transmitted LncRNA CATED Promotes Platinum-Resistance in High-Grade Serous Ovarian Cancer. Adv. Sci..

[108] Wambecke A., Ahmad M., Lambert B., Joly F., Poulain L., Denoyelle C., Meryet-Figuiere M. (2020). The Influence of Long Non-Coding RNAs on the Response to Chemotherapy in Ovarian Cancer. Gynecol. Oncol..

[109] Basu S., Nadhan R., Dhanasekaran D.N. (2025). Long Non-Coding RNAs in Ovarian Cancer: Mechanistic Insights and Clinical Applications. Cancers.

[110] Lu T.X., Rothenberg M.E. (2018). MicroRNA. J. Allergy Clin. Immunol..

[111] Zhang H., Lu B. (2020). MicroRNAs as Biomarkers of Ovarian Cancer. Expert Rev. Anticancer Ther..

[112] Weber J.A., Baxter D.H., Zhang S., Huang D.Y., Huang K.H., Lee M.J., Galas D.J., Wang K. (2010). The MicroRNA Spectrum in 12 Body Fluids. Clin. Chem..

[113] Ferreira P., Roela R.A., Lopez R.V.M., Del Pilar Estevez-Diz M. (2020). The Prognostic Role of MicroRNA in Epithelial Ovarian Cancer: A Systematic Review of Literature with an Overall Survival Meta-Analysis. Oncotarget.

[114] Yang D., Sun Y., Hu L., Zheng H., Ji P., Pecot C.V., Zhao Y., Reynolds S., Cheng H., Rupaimoole R. (2013). Integrated Analyses Identify a Master MicroRNA Regulatory Network for the Mesenchymal Subtype in Serous Ovarian Cancer. Cancer Cell.

[115] Li J., Tu Y., Wen J., Yao F., Wei W., Sun S. (2010). Role for Ezrin in Breast Cancer Cell Chemotaxis to CCL5. Oncol. Rep..

[116] Li S., Liu X., Zhou Y., Acharya A., Savkovic V., Xu C., Wu N., Deng Y., Hu X., Li H. (2018). Shared Genetic and Epigenetic Mechanisms between Chronic Periodontitis and Oral Squamous Cell Carcinoma. Oral Oncol..

[117] Hassan M.K., Waly A.A., Elsayed W., Keshk S., Allam W.R., El-khamisy S.F. (2021). Integrative MicroRNA and Gene Expression Analysis Identifies New Epigenetically Regulated MicroRNAs Mediating Taxane Resistance in Ovarian Cancer. Sci. Rep..

[118] Chhatriya B., Mukherjee M., Ray S., Sarkar P., Chatterjee S., Nath D., Das K., Goswami S. (2019). Comparison of Tumour and Serum Specific MicroRNA Changes Dissecting Their Role in Pancreatic Ductal Adenocarcinoma: A Meta-Analysis. BMC Cancer.

[119] Cochrane D.R., Spoelstra N.S., Howe E.N., Nordeen S.K., Richer J.K. (2009). MicroRNA-200c Mitigates Invasiveness and Restores Sensitivity to Microtubule-Targeting Chemotherapeutic Agents. Mol. Cancer Ther..

[120] Zhan X., Dong C., Liu G., Li Y., Li Y. (2017). Panel of Seven Long Noncoding RNA as a Candidate Prognostic Biomarker for Ovarian Cancer. Onco. Targets. Ther..

[121] Kujawa K.A., Lisowska K.M. (2015). Rak jajnika—od biologii do kliniki [Ovarian cancer—From biology to clinic]. Postepy Hig. Med. Dosw..

[122] Khabaz M.N., Qureshi I.A., Al-Maghrabi J.A. (2019). GLUT 1 Expression Is a Supportive Mean in Predicting Prognosis and Survival Estimates of Endometrial Carcinoma. Ginekol. Pol..

[123] Bhaskaran K., Douglas I., Forbes H., dos-Santos-Silva I., Leon D.A., Smeeth L. (2014). Body-Mass Index and Risk of 22 Specific Cancers: A Population-Based Cohort Study of 5·24 Million UK Adults. Lancet.

[124] Tworoger S.S., Huang T. (2016). Obesity and Ovarian Cancer. Obesity and Cancer.

[125] Huang T., Tworoger S.S., Willett W.C., Stampfer M.J., Rosner B.A. (2019). Associations of Early Life and Adulthood Adiposity with Risk of Epithelial Ovarian Cancer. Ann. Oncol..

[126] Ding N., Zhan J., Shi Y., Qiao T., Li P., Zhang T. (2022). Obesity in Children and Adolescents and the Risk of Ovarian Cancer: A Systematic Review and Dose-response Meta-Analysis. PLoS ONE.

[127] Li J., Han X. (2018). Adipocytokines and Breast Cancer. Curr. Probl. Cancer.

[128] Zwick R.K., Guerrero-Juarez C.F., Horsley V., Plikus M.V. (2018). Anatomical, Physiological, and Functional Diversity of Adipose Tissue. Cell Metab..

[129] Stępień S., Olczyk P., Gola J., Komosińska-Vassev K., Mielczarek-Palacz A. (2023). The Role of Selected Adipocytokines in Ovarian Cancer and Endometrial Cancer. Cells.

[130] Wang Y.Y., Hung A.C., Lo S., Yuan S.S.F. (2021). Adipocytokines Visfatin and Resistin in Breast Cancer: Clinical Relevance, Biological Mechanisms, and Therapeutic Potential. Cancer Lett..

[131] Pang L., Chang X. (2021). Resistin Expression in Epithelial Ovarian Cancer Promotes the Proliferation and Migration of Ovarian Cancer Cells to Worsen Prognosis. J. Cancer.

[132] Onstad M.A., Schmandt R.E., Lu K.H. (2016). Addressing the Role of Obesity in Endometrial Cancer Risk, Prevention, and Treatment. J. Clin. Oncol..

[133] Terzic M., Aimagambetova G., Kunz J., Bapayeva G., Aitbayeva B., Terzic S., Laganà A.S. (2021). Molecular Basis of Endometriosis and Endometrial Cancer: Current Knowledge and Future Perspectives. Int. J. Mol. Sci..

[134] Pereira S., Cline D.L., Glavas M.M., Covey S.D., Kieffer T.J. (2021). Tissue-Specific Effects of Leptin on Glucose and Lipid Metabolism. Endocr. Rev..

[135] Achkar I.W., Bhat A.A., Zafar M., Abbas M., Khan O.S., Kuttikrishnan S., Prabhu K.S., Uddin S. (2019). Oncogenic Role of Dysregulated Leptin Signaling in the Pathogenesis of Ovarian Cancer. Transl. Med. Commun..

[136] Bocian-Jastrzębska A., Malczewska-Herman A., Kos-Kudła B. (2023). Role of Leptin and Adiponectin in Carcinogenesis. Cancers.

[137] Nurgalieva A.K., Popov V.E., Skripova V.S., Bulatova L.F., Savenkova D.V., Vlasenkova R.A., Safina S.Z., Shakirova E.Z., Filonenko V.V., Bogdanov M.V. (2021). Sodium-dependent phosphate transporter NaPi2b as a potential predictive marker for targeted therapy of ovarian cancer. Biochem. Biophys. Rep..

